# A Noncanonical Protein Degradation Pathway That Regulates Germ Cell Maintenance

**DOI:** 10.1111/cpr.70259

**Published:** 2026-07-14

**Authors:** Meng Liu, Xiya Qiu, Wenying Qu, Yue Ma, Mingyuan Bao, Yuxuan Feng, Xiaochu Wang, Tao Zhou, Bo Zheng, Shunyu Hou, Yu Xia, Qingxia Meng

**Affiliations:** ^1^ Department of Obstetrics and Gynaecology The Affiliated Suzhou Hospital of Nanjing Medical University, Suzhou Municipal Hospital, Gusu School, Nanjing Medical University Suzhou China; ^2^ State Key Laboratory of Reproductive Medicine and Offspring Health, Center for Reproduction and Genetics The Affiliated Suzhou Hospital of Nanjing Medical University, Suzhou Municipal Hospital, Gusu School, Nanjing Medical University Suzhou China; ^3^ Department of Urology The Affiliated Suzhou Hospital of Nanjing Medical University, Suzhou Municipal Hospital, Gusu School, Nanjing Medical University Suzhou China; ^4^ State Key Laboratory of Reproductive Medicine and Offspring Health, Department of Histology and Embryology School of Basic Medical Sciences, Nanjing Medical University Nanjing China; ^5^ Scientific Research Center Gannan Medical University Ganzhou China

## Abstract

SENP8 regulates a non‐canonical NEDD8‐dependent pathway to stabilize STAT1, promote CCL2/CCL5 transcription, and maintain germ cell proliferation and migration.

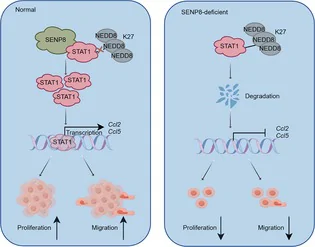

To the Editor,

Maintaining cellular and organismal homeostasis relies on the dynamic balance between protein synthesis and degradation [1]. In eukaryotic systems, protein degradation is primarily executed through two canonical pathways—the ubiquitin–proteasome system (UPS) and the autophagy–lysosome pathway—which cooperate to preserve proteome integrity by eliminating misfolded, damaged or superfluous proteins [2]. Traditionally, proteasomal degradation has been considered predominantly ubiquitin‐dependent. However, recent reports have expanded this view by identifying additional proteolytic mechanisms, including the recently described midnolin–proteasome pathway, which mediates ubiquitin‐independent substrate delivery to the proteasome [3]. These emerging noncanonical degradation routes suggest that proteostasis can be regulated through alternative substrate‐recognition and clearance mechanisms beyond the classical UPS paradigm. Despite these advances, how posttranslational modifiers interface with proteostasis networks to safeguard adult germ cell populations, such as spermatogonia, remains insufficiently understood.

The UPS plays a central role in maintaining protein homeostasis and regulating spermatogenesis [4], while ubiquitin‐like (UBL) modifiers add further regulatory layers by modulating protein function rather than directing their destruction [5]. NEDD8, a ubiquitin‐like modifier and paralog of ubiquitin, differs from ubiquitin in that it primarily modulates the activity, conformation or interaction landscape of target proteins rather than marking them for degradation [6]. The best‐characterized substrates of NEDD8 are cullin family scaffold proteins, whose neddylation is required for the activation of cullin–RING ligases (CRLs), thereby influencing diverse signalling outputs [6]. Beyond cullins, emerging evidence indicates that NEDD8 also regulates noncullin targets to fine‐tune cell proliferation, stress adaptation, DNA repair and developmental programmes [7]. In the testis, accumulating evidence suggests that neddylation contributes to meiotic progression and spermatogenic differentiation, and large‐scale proteomics has revealed widespread neddylation in spermatocytes [8]. Given the tight coupling between NEDD8 conjugation and intracellular signalling pathways [9], it remains unclear whether NEDD8 directly modulates protein degradation to influence these pathways. Likewise, whether neddylation or its reversal contributes to the regulation of germ cells also remains largely unknown.

SENP8, also known as NEDP1, is the principal deneddylase responsible for removing NEDD8 from cullin and noncullin substrates, thereby maintaining neddylation homeostasis [10]. Loss of SENP8 leads to a pronounced accumulation of intracellular NEDD8 [11], perturbing SENP8 offers a practical strategy to interrogate NEDD8‐dependent regulatory mechanisms. Given the essential role of proteostasis in cell maintenance, we hypothesized that SENP8 safeguards germ cell homeostasis through posttranslational regulatory mechanisms associated with protein quality control.

Building on this rationale, in this study, we identify a noncanonical protein degradation pathway in germ cells involving SENP8‐mediated de‐neddylation. We show that loss of SENP8 markedly compromises proliferative capacity and perturbs key transcriptional programmes associated with germ cell activity. Mechanistically, SENP8 maintains the stability of STAT1 by preventing its aberrant neddylation and subsequent degradation, thereby enabling transcriptional activation of the chemokines *Ccl2* and *Ccl5*. *Ccl5* depletion, but not *Ccl2* depletion, reduced STAT1 phosphorylation without markedly affecting total STAT1 levels, suggesting a potential link between CCL5 signalling and STAT1 activation in germ cells. Our study challenges the traditional functional boundary of neddylation and establishes a novel regulatory axis essential in germ cells, with broader implications for understanding noncanonical protein degradation systems.

## 1 Results

### 1.1 SENP8 Is Essential for the Proliferation and Migratory Capacity of Germ Cells

The proliferation and motility of germ cells are critical for sustaining spermatogenesis and ensuring continuous sperm output [12]. To investigate the biological function of SENP8 in germ cell regulation, we employed the mouse spermatogonial cell line GC1 as a model. RT‐qPCR confirmed that transfection with two independent siRNAs efficiently reduced *Senp8* expression compared with the negative control group (Figure 1A). Cell Counting Kit‐8 (CCK‐8) assays demonstrated that *Senp8* knockdown significantly suppressed the proliferation rate of GC1 cells across multiple time points (Figure 1B). Consistent with this observation, the colony formation assay revealed a marked decrease in both the number and size of colonies following *Senp8* depletion (Figure 1C,E). In addition, transwell migration analysis indicated that downregulation of *Senp8* evidently weakened the migratory capacity of GC1 cells (Figure 1D,F). Together, these findings highlight the potential role of *Senp8* in maintaining the homeostasis and regenerative capacity of the germ cell population.

**FIGURE 1:**
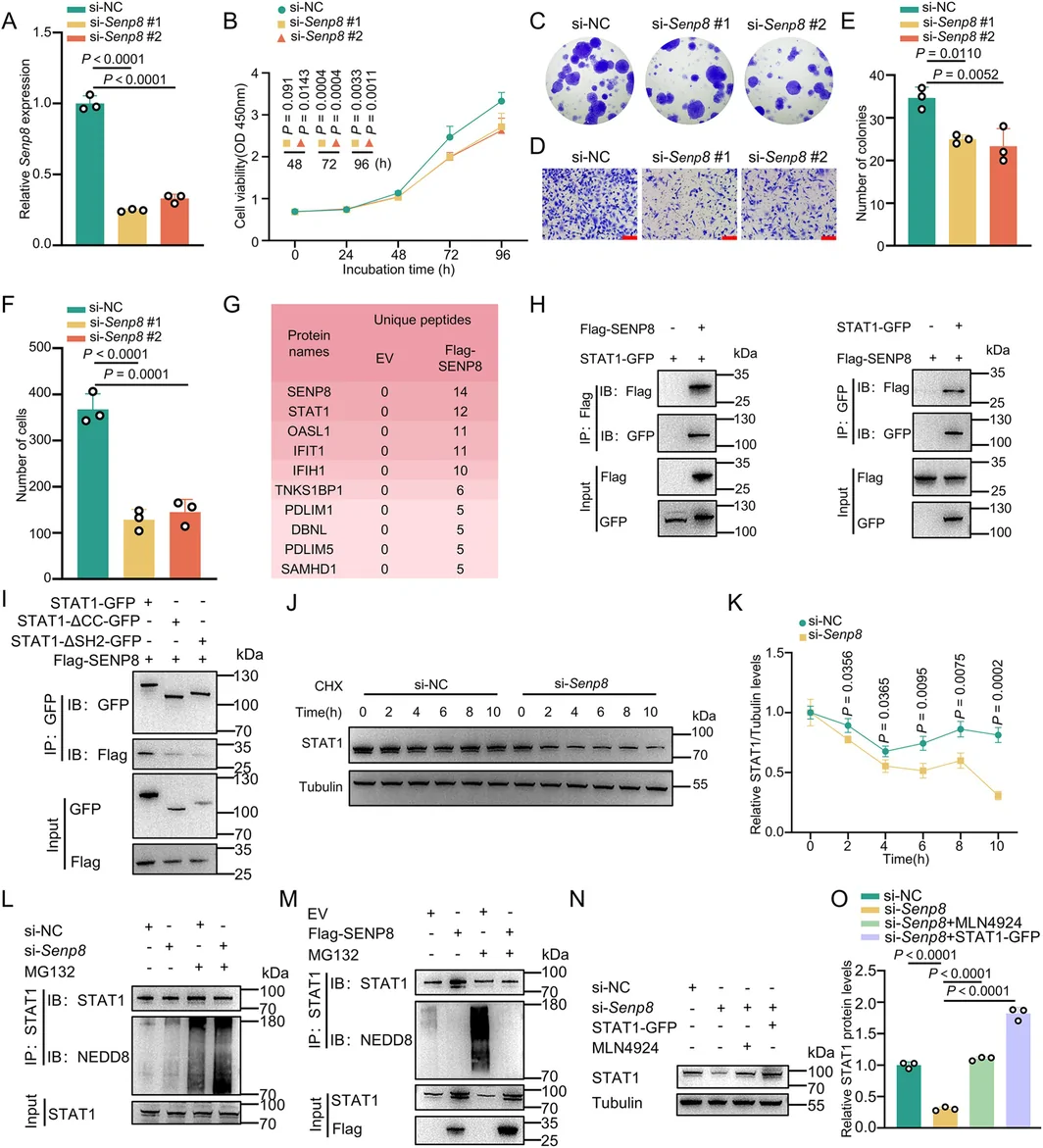
SENP8 maintains germ cell function by regulating STAT1 stability and activation through deneddylation. (A) *Senp8* mRNA level in GC1 cells transfected with control siRNA (si‐NC) or *Senp8*‐targeting siRNA (si‐*Senp8*), *n* = 3 per group. (B) Cell counting kit‐8 (CCK8) assay of GC1 proliferation after *Senp8* knockdown, *n* = 6 per group. (C and E) Colony formation assays were performed to assess the proliferative ability of *Senp8*‐knockdown GC1 cells, *n* = 3 per group. (D and F) Transwell experiments showing reduced migration in *Senp8*‐knockdown GC1 cells, *n* = 3 per group. Scale bar = 100 μm. (G) Top 10 ranked proteins by peptide number identified in the mass spectrometry results. (H) Reciprocal IP assays of GC1 cells lysated with anti‐Flag and anti‐GFP beads, followed by immunoblotted (IB) with the specified antibodies. (I) Identification of the STAT1 region essential for SENP8 binding using domain‐specific mutations. (J and K) Western blotting experiments showed the protein expression of STAT1 by GC1 cells transfected with si‐*Senp8* and treated for specified times with cycloheximide (CHX, 100 μg/mL), *n* = 3 per group. (L) STAT1 neddylation in GC1 cells after *Senp8* knockdown (si‐*Senp8*) versus control (si‐NC), detected by anti‐NEDD8 immunoblotting (IB) of STAT1 immunoprecipitates (IPs) from MG132 (20 μM, 6 h)‐treated cells. (M) STAT1 neddylation in GC1 cells overexpressing SENP8 (Flag‐SENP8), detected as in A. (N) STAT1 levels in GC1 cells under the following conditions: si‐*Senp8*, si‐*Senp8* + MLN4924 (a selective NEDD8‐activating enzyme (NAE) inhibitor), and si‐*Senp8* + STAT1 overexpression. (O) Statistical chart of N. *n* = 3 per group. Each experiment was independently repeated three times. The relevant *p* values had been marked in the figures.

To elucidate the molecular partners through which SENP8 exerts its regulatory role in germ cells, we performed co‐immunoprecipitation (IP) coupled with LC–MS/MS analysis in GC1 cells transfected with Flag‐tagged SENP8 (Figure S1A). Mass spectrometry identified several potential SENP8‐interacting proteins, among which STAT1 emerged as one of the most enriched candidates, showing the top 10 genes with unique peptides specifically detected in the Flag‐SENP8 immunoprecipitates (Figure 1G). To validate this interaction, reciprocal co‐IP assays were carried out in GC1 cells co‐expressing Flag‐SENP8 and STAT1‐GFP. As shown in Figure 1H, STAT1 was efficiently co‐precipitated with SENP8, and vice versa, confirming a specific association between these two proteins.

We next sought to map the key domains on STAT1 required for this interaction. Based on the protein structure of STAT1 [13], deletion mutants lacking the coiled‐coil (ΔCC, residues 136–317) or SH2 (ΔSH2, residues 573–670) domains were generated (Figure S1B). These mutants, or wild‐type STAT1‐GFP, were co‐transfected with Flag‐SENP8 for Co‐IP analysis. As shown in Figure 1I, wild‐type STAT1 efficiently interacted with Flag‐SENP8, whereas deletion of either the coiled‐coil domain (STAT1‐ΔCC‐GFP) or the SH2 domain (STAT1‐ΔSH2‐GFP) markedly reduced this interaction. Taken together, these results clearly demonstrate that STAT1 is a novel interacting partner of SENP8 in germ cells, and this interaction indispensably requires both the Coiled‐Coil and SH2 domains of STAT1. This finding lays the groundwork for our subsequent investigation into how SENP8 regulates STAT1 stability.

### 1.2 SENP8 Maintains STAT1 Activation and Stability via Deneddylation in Germ Cells

To define the functional contribution of STAT1 in germ cells, we confirmed efficient *Stat1* knockdown in GC‐1 cells by RT–qPCR and Western blot (Figure S1C–E). STAT1 depletion markedly compromised cell viability and clonogenic growth (Figure S1F–G,I), and also reduced migratory capacity in transwell assays (Figure S1H,J).

Given the physical association between SENP8 and STAT1, we next asked whether SENP8 modulates STAT1 stability and activation. *Senp8* knockdown decreased both total STAT1 and its phosphorylation at Tyr701 and Ser727, whereas SENP8 overexpression produced the opposite effect (Figure S1K–N), supporting a role for SENP8 in sustaining STAT1 signalling in germ cells.

To further validate this regulation beyond the GC‐1 cell line, we examined the effects of *SENP8* knockdown in human SSCs established in our previous study [14]. Consistently, *SENP8* depletion reduced STAT1 protein abundance (Figure S2A–C), impaired cell viability and migration in human SSCs (Figure S2D–F), and increased STAT1 NEDD8 modification in human SSCs (Figure S2G).

To assess cell‐type specificity, we examined *Senp8* silencing in TM3 Leydig cells (Figure S3A). In contrast to GC‐1 cells, *Senp8* knockdown did not affect STAT1 protein abundance (Figure S3B,C), although it still impaired proliferation and migration (Figure S3D–H).

Together, these results indicate that SENP8 regulates proliferative and migratory phenotypes in testicular cell models, but selectively supports STAT1 stability in germ cells.

SENP8 has been characterized primarily as a regulator of cullin neddylation, functioning through nonproteolytic mechanisms that indirectly modulate CRL activity [10]. To determine whether SENP8 affects STAT1 protein stability, we performed cycloheximide (CHX) chase assays to measure the half‐life of STAT1. Unexpectedly, *Senp8* depletion accelerated STAT1 degradation (Figure 1J,K), suggesting that SENP8 exerts a stabilising effect on STAT1 protein in germ cells. These findings uncover a previously unappreciated role of SENP8 in stabilising STAT1, suggesting that neddylation exerts direct control over transcription factor homeostasis.

SENP8 has been reported to selectively interact with NEDD8 and to contribute to its proteolytic maturation [10]. Because SENP8 depletion reduced STAT1 abundance, we asked whether SENP8 controls STAT1 stability through NEDD8 conjugation. SENP8 knockdown markedly increased endogenous STAT1 neddylation, whereas SENP8 overexpression produced the opposite effect (Figure 1L,M). This regulation was recapitulated in an exogenous system co‐expressing Myc‐NEDD8 and GFP‐STAT1 (Figure S4A,B). Treatment with the proteasome inhibitors MG132 and Epoxomicin both led to the accumulation of NEDD8‐modified STAT1, supporting an association between STAT1 NEDDylation and proteasome‐dependent turnover (Figures 1L,M and S4C,D). To further identify the potential NEDDylation site, we generated a series of lysine‐to‐arginine STAT1 mutants (Figure S4E). Among them, K240R markedly reduced STAT1 NEDDylation and partially rescued the decrease in cell viability and proliferation caused by *Senp8* knockdown (Figure S4E,H). These results suggest that K240 is a key NEDDylation site through which SENP8 may regulate STAT1 stability in germ cells.

To further assess the functional consequence of NEDD8 modification on STAT1 stability, we compared the effects of *Senp8* silencing, pharmacological NEDD8 inhibition using MLN4924, and exogenous STAT1 expression. Both MLN4924 treatment and STAT1 overexpression partially restored STAT1 protein levels reduced by *Senp8* knockdown (Figure 1N,O). Since MLN4924 broadly inhibits NEDD8 activation, we further examined representative CRL substrates, including p27 and NRF2, and assessed STAT1 ubiquitination. Neither p27 nor NRF2 accumulation nor marked changes in STAT1 ubiquitination were observed after *Senp8* knockdown or MLN4924 treatment (Figure S4I–K). These results suggest that SENP8 may protect STAT1 stability through a NEDD8‐associated mechanism, without detectably affecting canonical CRL activity or STAT1 ubiquitination in germ cells.

Given that NEDD8 can form poly‐chains through multiple lysine residues [11], we next sought to define the linkage specificity by which SENP8 regulates STAT1. To this end, a panel of lysine‐restricted NEDD8 mutants was employed to individually permit distinct chain types. We found that STAT1 neddylation was specifically supported by K27‐linked NEDD8 chains: both wild‐type NEDD8 and the K27‐retaining mutant generated robust NEDD8‐STAT1 conjugates, whereas constructs restricting other linkage types produced minimal modification under identical conditions (Figure 2A). Notably, co‐expression of SENP8 markedly diminished K27‐linked NEDD8 conjugation on STAT1, establishing K27 as the principal linkage removed by SENP8.

**FIGURE 2:**
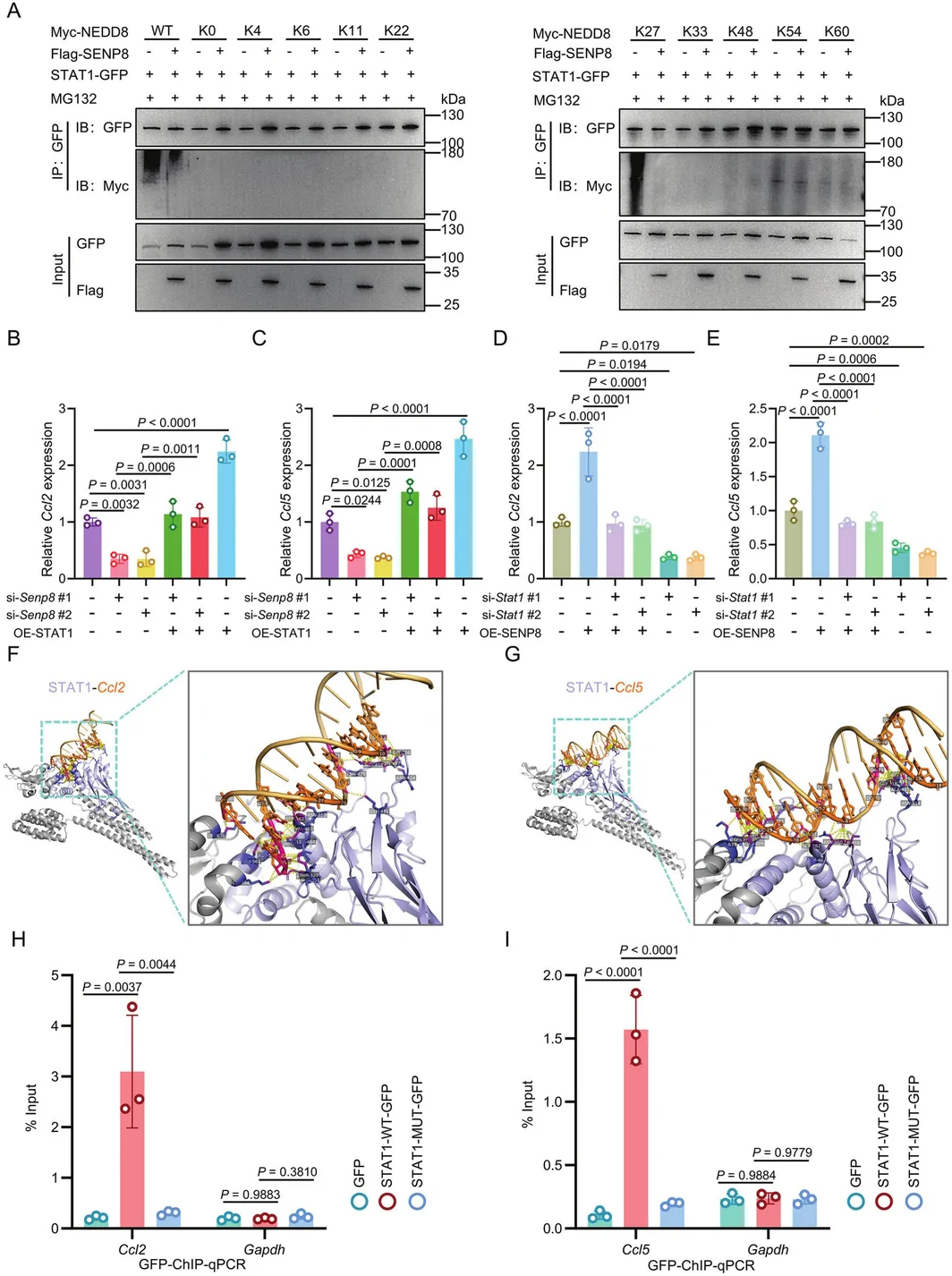
SENP8 regulates CCL2/CCL5 transcription and chemokine signalling through control of STAT1 in germ cells. (A) Co‐transfected Myc‐NEDD8 (WT, K0, K4, K6, K11, K22, K27, K33, K48, K54, K60), STAT1‐GFP and Flag‐SENP8 or EV plasmids in GC1 cells were subjected to Co‐IP assay, treated with MG132 (20 μM, 6 h). The level of STAT1 ubiquitination‐like modification was observed using Myc antibody. (B) The relative expression levels of *Ccl2* under different combinatorial conditions of *Senp8* knockdown and STAT1 overexpression. (C) The relative expression levels of *Ccl5* under different combinatorial conditions of Senp8 knockdown and STAT1 overexpression. (D) The relative expression levels of *Ccl2* under different combinatorial conditions of *Stat1* knockdown and SENP8 overexpression. (E) The relative expression levels of *Ccl5* under different combinatorial conditions of *Stat1* knockdown and SENP8 overexpression. For B‐E, *n* = 3 per group. (F) Structural model and interacting interface for STAT1‐*Ccl2*. (G) Structural model and interacting interface for STAT1‐*Ccl5*. (H) Chromatin immunoprecipitation coupled with quantitative polymerase chain reaction (ChIP‐qPCR) was conducted to analyse the DNA sequences corresponding to the putative STAT1‐WT‐GFP and STAT1‐MUT‐GFP binding regions within the *Ccl2* promoter in GC1 cells. The *Gapdh* gene served as a negative control. (I) ChIP‐qPCR was conducted to analyse the DNA sequences corresponding to the putative STAT1‐WT‐GFP and STAT1‐MUT‐GFP binding regions within the *Ccl5* promoter in GC1 cells. The *Gapdh* gene served as a negative control. For H‐I, *n* = 3 per group. Each experiment was independently repeated three times. The relevant *p* values had been marked in the figures.

Together, these findings identify STAT1 as a K27‐linked NEDD8 substrate and establish SENP8 as a linkage‐specific deneddylase that preserves STAT1 stability by preventing its proteasome‐dependent degradation.

### 1.3 SENP8–STAT1 Axis Regulates CCL2/CCL5 Expression and Chemokine Signalling Responses in Germ Cells

To identify downstream effectors of the SENP8–STAT1 axis, we performed RNA‐seq in GC‐1 cells after *Senp8* or *Stat1* knockdown with three biological replicates per group (Figure S5A). Differential gene expression analysis was performed by comparing the si‐*Senp8* or si‐*Stat1* groups with the si‐NC control group. *p* values were adjusted using the Benjamini–Hochberg method, and genes with an absolute log2 fold change greater than 0.65 and an adjusted *p* value below 0.05 were defined as differentially expressed genes (DEGs). Intersection of the two DEG lists yielded 40 shared genes (Figure S5B–D), among which *Ccl2* and *Ccl5* emerged as consistently downregulated candidates (Figure S5E,F). Given that *Ccl2/Ccl5* have been reported as STAT1‐responsive genes, we prioritized them for validation. Given that *Ccl2* and *Ccl5* are established transcriptional targets of STAT1 [15, 16], we focused on these candidates for functional validation.

To functionally validate these candidates, we individually silenced *Ccl2* and *Ccl5* in GC‐1 cells (Figure S6A–C,I–K) and found that depletion of either chemokine markedly impaired cell proliferation and cell motility (Figure S6D–H,L–P), phenocopying the defects observed upon SENP8 and STAT1 depletion.

Mechanistically, *Senp8* knockdown markedly decreased *Ccl2/Ccl5* transcripts, whereas STAT1 overexpression restored their expression (Figure 2B,C). Conversely, SENP8 overexpression upregulated *Ccl2/Ccl5*, an effect largely blunted by *Stat1* silencing (Figure 2D,E). Together, these data support that SENP8 promotes *Ccl2/Ccl5* transcription in a STAT1‐dependent manner, placing these chemokines downstream of the SENP8–STAT1 pathway.

To further define the transcriptional mechanism, we predicted STAT1‐binding elements within the *Ccl2* and *Ccl5* promoters. Putative motifs were highly enriched within ±2 kb of the transcription start sites, consistent with proximal promoter engagement (Figure S7A). Molecular docking analyses further indicated that the top‐ranked motifs of both promoters formed stable complexes with the DNA‐binding region (DBR) of STAT1 (Figures 2F,G and S7B–D).

Interface mapping further identified seven residues—ASN414, GLY416, ASN417, ARG418, ASN460, SER462 and LYS636—shared between the STAT1–*Ccl2* and STAT1–*Ccl5* complexes (Figure S7D). Notably, six of these residues are located within the DBR of STAT1, highlighting a conserved structural interface mediating dual promoter recognition. Collectively, these data demonstrate that SENP8‐activated STAT1 directly binds to the promoter regions of *Ccl2* and *Ccl5* through conserved DBR residues, thereby enhancing their transcription to affect the germ cells.

### 1.4 STAT1 Regulates *Ccl2* and *Ccl5* Transcription Through Promoter Binding

To determine whether STAT1 regulates *Ccl2* and *Ccl5* transcription through promoter binding, we first compared wild‐type STAT1 with a DNA‐binding–defective mutant. Overexpression of wild‐type STAT1 (STAT1‐WT‐GFP) increased *Ccl2/Ccl5* mRNA levels in a dose‐dependent manner, whereas the DNA‐binding mutant (STAT1‐MUT‐GFP) completely lost this activity (Figure S7E,F), indicating that STAT1‐mediated induction of these chemokines depends on its DNA‐binding capacity.

Luciferase reporter assays further demonstrated that STAT1‐WT strongly activated the wild‐type promoters of *Ccl2* and *Ccl5*, but not the promoters in which the STAT1‐binding motifs were disrupted (promoter‐MUT). Similarly, mutation of STAT1's DNA‐binding domain (STAT1‐MUT) abolished promoter activation, confirming that STAT1 directly engages these promoter elements (Figure S7G,H). To further define the STAT1 residues involved in promoter activation, we selected six candidate sites based on preliminary prediction of the STAT1 DBR and generated individual STAT1 mutant constructs. Among the tested mutants, alterations at residues 414 and 417 markedly weakened STAT1‐mediated activation of both *Ccl2* and *Ccl5* promoter reporters (Figure S8A,B).

Chromatin immunoprecipitation (ChIP–qPCR) provided in situ validation: STAT1‐WT‐GFP was significantly enriched at the promoter regions of *Ccl2* and *Ccl5*, but not at the *Gapdh* negative control locus (Figure 2H,I). Collectively, these data demonstrate that STAT1 directly binds to the promoters of *Ccl2* and *Ccl5* via its DNA‐binding domain to activate transcription. This mechanistic link connects SENP8‐mediated stabilisation of STAT1 to the precise transcriptional control of key chemokines governing germ cell proliferation and migration.

Given reports in immune cells that CCL5 can enhance STAT1 phosphorylation to reinforce M1 macrophage polarisation [16], we asked whether CCL5 is associated with STAT1 activation in germ cells. Silencing *Ccl2* had no effect on total STAT1 or its phosphorylation, whereas *Ccl5* depletion reduced STAT1 phosphorylation at Tyr701 and Ser727 without altering total STAT1 levels (Figure S9A–D). These results suggest that CCL5 may contribute to maintaining STAT1 activation in this system.

Together, these findings support a potential SENP8–STAT1–CCL2/CCL5 regulatory relationship in germ cells (Figure S9E).

## 2 Discussion

In this study, we reveal a germ cell‐associated SENP8–STAT1 regulatory axis that links deneddylation to STAT1 stability and chemokine expression. In germline‐derived cells, SENP8 depletion reduced STAT1 abundance, impaired proliferation and in vitro migratory capacity, and disrupted STAT1‐dependent *Ccl2*/*Ccl5* expression. By contrast, *Senp8* knockdown in TM3 Leydig cells affected cellular phenotypes without decreasing STAT1 protein levels. This distinction suggests that SENP8‐dependent control of STAT1 stability represents a germ cell‐biased regulatory mechanism rather than a universal consequence of SENP8 loss. These insights not only expand the biological scope of neddylation‐related enzymes but also connect immunoregulatory chemokines to germ cell maintenance.

Neddylation is widely recognized as a ubiquitin‐like modification that predominantly modulates protein conformation, subcellular localisation and enzymatic activity rather than promoting proteasomal degradation [9, 17]. Consistent with this view, previous studies established NEDD8 as an essential regulator of cullin‐RING ligase (CRL) activation, thereby indirectly shaping protein turnover through CRL‐mediated ubiquitination rather than serving as a degradation signal itself [6, 7]. A notable finding of our study is that SENP8, the principal deneddylase, directly safeguards STAT1 protein stability through a mechanism fundamentally distinct from its canonical role in regulating cullin neddylation. Also, we identify SENP8 as a key regulator of germ cells and demonstrate that its loss impairs germ cell proliferation, cell motility and survival, indicating that proper neddylation homeostasis is essential for maintaining germ cell function.

STAT1 is best known for its roles in cytokine signalling [13], yet how its activity is maintained in germ cells has been unclear. We show that STAT1 is modified by NEDD8 and that SENP8 loss markedly enhances this modification, accelerating STAT1 degradation. Mechanistically, we identify K27‐linked NEDD8 chains as the functional signal mediating STAT1 turnover—an unprecedented observation, given that NEDD8 chains have not previously been implicated in proteasomal targeting. The ability of proteasome inhibitor MG132 and Epoxomicin to accumulate the neddylated STAT1 protein levels further demonstrates that the proteasome can degrade NEDD8‐modified substrates, extending the classical view that it primarily recognizes K48‐linked ubiquitin chains [2, 18, 19].

As a transcription factor, STAT1 promoted *Ccl2* and *Ccl5* expression through promoter‐associated binding. Interestingly, CCL5 was associated with the maintenance of STAT1 phosphorylation, suggesting that CCL5 may be linked to the maintenance of STAT1 phosphorylation in germ cells. Nevertheless, the upstream receptor and kinase events connecting CCL5 to STAT1 phosphorylation remain to be further clarified.

Together, our findings suggest that SENP8 protects STAT1 stability through a NEDD8‐associated regulatory mechanism in germ cells. STAT1 NEDDylation, including K27‐linked NEDD8 chain formation, was associated with reduced STAT1 abundance, whereas SENP8 limited this modification and maintained STAT1 protein levels. Downstream, STAT1 promoted *Ccl2* and *Ccl5* transcription and CCL5 may be linked to sustained STAT1 phosphorylation. This SENP8–STAT1 regulatory relationship was observed in germline‐derived cells but not in TM3 Leydig cells, suggesting potential cell‐type specificity. This germline‐specific deneddylation axis provides insight into NEDD8‐related protein regulation in germ cell biology. Although our study revealed an interesting SENP8–STAT1 regulatory relationship in germline‐derived cells, the current findings are primarily based on in vitro cell models, and the precise in vivo mechanisms and physiological relevance of this axis in the testis require further investigation.

## 3 Materials and Methods

### 3.1 Cell Culture and Treatment

The GC‐1 cell line, derived from mouse spermatogonia, was obtained from the American Type Culture Collection (Manassas, VA, USA). Cells were maintained in Dulbecco's Modified Eagle Medium (DMEM; Meilun Bio, China) supplemented with 10% foetal bovine serum (FBS; TransGen Biotech, China) and 1% penicillin–streptomycin (PS, NCM Biotech, China). Cultures were incubated in a humidified atmosphere at 37°C with 5% CO_2_.

TM3 cells were maintained in DMEM/F‐12 (Meilun Bio, China) supplemented with 2.5% FBS, 5% HI Bovine Serum (HS; gibco, USA) and 1% PS. Cultures were incubated in a humidified atmosphere at 37°C with 5% CO_2_.

The human Spermatogonial stem cell (SSC) line was kindly provided by Professor Zuping He at Hunan Normal University (China) [14]. Cells were maintained in DMEM/F‐12 supplemented with 10% FBS (Sigma, Germany) and 1% PS. Cultures were incubated in a humidified atmosphere at 37°C with 5% CO_2_.

### 3.2 Cell Transfection

Cells were transfected with specific siRNAs (GenePharma, China; Tsingke Biotech, China) when confluency reached 60%–80%, using lipofectamine 2000 (Invitrogen, USA) as the transfection reagent. The sequences of the siRNAs used are listed below:

si‐NC 5′‐UUCUCCGAACGUGUCACGUTT‐3′

si‐*Senp8* #1 5′‐GCAAUCAGAUGUCUCACUATT‐3′

si‐*Senp8* #2 5′‐GCAGAGAAACUGAAGGCUUTT‐3′

si‐*Stat1* #1 5′‐CUGCCUAUGAUGUCUCGUUTT‐3′

si‐*Stat1* #2 5′‐GGACGUUCCUGCUUAGAUUTT‐3′

si‐*Ccl2* #1 5′‐GUGAAGUUGACCCGUAAAU‐3′

si‐*Ccl2* #2 5′‐CCGUAAAUCUGAAGCUAAU‐3′

si‐*Ccl5* #1 5′‐GUCAAGGAGUAUUUCUACA‐3′

si‐*Ccl5* #2 5′‐CUUGCAGUCGUGUUUGUCA‐3′

si‐*SENP8* #1 5′‐GCGGCAAUCAGAUGUCUCA‐3′

si‐*SENP8* #2 5′‐UGACCAUAUUAUUGGGUUU‐3′

According to the manufacturer's instructions, overexpression plasmids were delivered into cells using X‐treme GENE HP DNA Transfection Reagent (Roche, Switzerland). All plasmid constructs were designed and generated by Sangon Biotech Inc. (Shanghai, China). The plasmids used in this study included:

pcDNA3.1‐Flag‐mouse‐SENP8, pEGFP‐N1‐mouse‐STAT1

pEGFP‐N1‐mouse‐STAT1‐ΔCC, pEGFP‐N1‐mouse‐STAT1‐ΔSH2

pEGFP‐N1‐mouse‐STAT1‐K240R, pEGFP‐N1‐mouse‐STAT1‐K379R

pEGFP‐N1‐mouse‐STAT1‐K592R, pEGFP‐N1‐mouse‐STAT1‐K636R

pEGFP‐N1‐mouse‐STAT1‐K637R, pEGFP‐N1‐mouse‐STAT1‐K652R

pEGFP‐N1‐mouse‐STAT1‐K673R, pEGFP‐N1‐mouse‐STAT1‐K685R

pcDNA3.1‐Myc‐NEDD8, pcDNA3.1‐Myc‐NEDD8‐K0

pcDNA3.1‐Myc‐NEDD8‐K4, pcDNA3.1‐Myc‐NEDD8‐K6

pcDNA3.1‐Myc‐NEDD8‐K11, pcDNA3.1‐Myc‐NEDD8‐K22

pcDNA3.1‐Myc‐NEDD8‐K27, pcDNA3.1‐Myc‐NEDD8‐K33

pcDNA3.1‐Myc‐NEDD8‐K48, pcDNA3.1‐Myc‐NEDD8‐K54

pcDNA3.1‐Myc‐NEDD8‐K60

In experiments using MG132 (a proteasome inhibitor) and Epoxomicin (a proteasome inhibitor), cells were initially transfected for 48 h and then treated with 20 μM MG132 or 2 μM Epoxomicin for 6 h prior to further experiments.

### 3.3 RNA Extraction and Reverse‐Transcription Quantitative PCR (RT‐qPCR)

Total RNA was isolated from treated cells using Trizol reagent (Vazyme, China), followed by cDNA synthesis with reverse transcription reagents (Cat. R323‐01, Vazyme, China). The relative mRNA expression levels were quantified using Taq Pro Universal SYBR qPCR Master Mix (Vazyme, China) on an Applied Biosystems 7500 Real‐Time PCR System. The corresponding primer sequences are listed below:

18 s RNA‐F:5′‐AAACGGCTACCACATCCAAG‐3′

18 s RNA‐R: 5′‐CCTCCAATGGATCCTCGTTA‐3′

*Senp8*‐F:5′‐CAAGCGTAGGGACACAGGTT‐3′

*Senp8*‐R: 5′‐GGGGAGACAGGCTGATGTTT‐3′

*Stat1*‐F:5′‐TCGTGGGTTGGTTCTTCTGG‐3′

*Stat1*‐R: 5′‐TGTGCACACTTACCGTGGTT‐3′

*Ccl2*‐F:5′‐AACTGCATCTGCCCTAAGGT‐3′

*Ccl2*‐R: 5′‐AGGCATCACAGTCCGAGTCA‐3′

*Ccl5*‐F:5′‐CACCATATGGCTCGGACACC‐3′

*Ccl5*‐R: 5′‐CTTGGCGGTTCCTTCGAGT‐3′

*SENP8*‐F:5′‐AATTTCTGAGCAGCCCTCGT‐3′

*SENP8*‐R:5′‐GGGCTGATGAAACTGACGTG‐3′

### 3.4 Cell Proliferation Assays

Following transfection, cells were seeded into 96‐well plates at a density of 2500 cells per well. Cell viability was assessed using the CCK8 (APEX × BIO) in accordance with the previously established protocol [20]. Absorbance was measured at 450 nm using a microplate reader (Bio‐Rad Model 680, USA) at 0, 24, 48, 72 and 96 h after seeding.

In the colony formation assay, 1000 treated cells were seeded per well into six‐well plates. After a 2‐week incubation, colonies were fixed with methanol and stained with crystal violet (Beyotime, China). The proliferative capacity was evaluated by counting the number of colonies formed.

### 3.5 Cell Migration Assays

Cell migration was assessed via Transwell assays conducted in 24‐well plates (8 μm pore size, Corning, USA). In brief, the upper chamber was loaded with 300 μL of serum‐free medium containing 45,000 cells, whereas the lower chamber was filled with 700 μL of complete medium to serve as a chemoattractant. Following a 48‐h incubation period, cells that had migrated to the lower chamber were fixed and stained following the protocol outlined in previous studies [14]. Images were then acquired under a microscope for subsequent quantitative evaluation.

### 3.6 IP and Liquid Chromatography‐Mass Spectrometry (LC–MS/MS) Analysis

The appropriate plasmids were transfected into GC1 cells. For exogenous IP, 48–72 h posttransfection, the cells were harvested and lysed using RIPA lysis buffer (NCM, China) supplemented with 1% phenylmethylsulfonyl fluoride (PMSF). Subsequently, the cell lysates were centrifuged to eliminate cellular debris, and the supernatants were carefully retrieved. To mitigate nonspecific protein binding, 20 μL of protein A/G magnetic beads (Vazyme, China) were added to each supernatant, followed by a 2‐h incubation at low speed. Subsequently, the supernatants were collected and incubated overnight (12–16 h) at 4°C with an appropriate quantity of anti‐FLAG nanobody agarose beads (AlpaLifeBio, China). On the following day, the immunoprecipitates were washed thrice with the lysis buffer. The eluted samples were then heated at 100°C for 10 min in 1× sodium dodecyl sulfate (SDS) buffer. The samples were electrophoresed on a SDS–PAGE and subsequently analysed using LC‐tandem mass spectrometry (LC–MS/MS) for the IP products, following the methodology described previously [21].

For endogenous (IP), after minimising nonspecific protein binding, the supernatant was collected and incubated with 5 μg of the corresponding primary antibody at 4°C overnight. On the following day, 50 μL of protein A/G magnetic beads (Vazyme, China) were added, and the mixture was incubated at 4°C for 3 h. Subsequently, the immunoprecipitates were washed three times with the lysis buffer. The subsequent experimental procedures were the same as described above.

### 3.7 Western Blotting

Cells were harvested and washed twice with prechilled PBS to minimize protein degradation. Cell lysis was performed using RIPA buffer (NCM, China) supplemented with 1% PMSF (Beyotime, China) as a protease inhibitor. Following centrifugation, the supernatant was collected, and protein concentration was determined using a BCA Protein Assay Kit (NCM, China). Proteins were then denatured by heating at 100°C for 10 min. Equal amounts of protein were separated via 10% SDS–polyacrylamide gel electrophoresis (SDS‐PAGE, GenScript, China) and subsequently transferred onto polyvinylidene difluoride (PVDF) membranes (Merck Millipore, USA). The membranes were blocked with 5% skimmed milk in TBST (Tris‐buffered saline containing 0.1% Tween‐20) at room temperature for 1 h to reduce nonspecific binding, followed by overnight incubation at 4°C with appropriate primary antibodies. On the following day, membranes were washed three times with TBST, incubated with corresponding secondary antibodies for 1 h at room temperature, and then washed again three times. Immunoreactive bands were visualized using an enhanced chemiluminescence (ECL) detection system, and band intensities were quantified using Image‐Pro Plus 6.0 software. The antibodies that were utilized comprised anti‐STAT1 (1:2000; 10,144‐2‐AP; Proteintech), anti‐NEDD8 (1:1000; 2745; CST), anti‐Tubulin (1:3000; Cat No. AT819; Beyotime), anti‐Flag (1:1000; F1804; Sigma), anti‐GFP (1:2000; ab290; Abcam) and anti‐Myc (1:1000; 2276; CST).

### 3.8 Protein Half‐Life Assay

GC1 cells were transfected with either si‐NC or si‐*Senp8* for a duration of 48–72 h. Following transfection, protein synthesis was blocked by treatment with CHX (100 μg/mL). Subsequently, total cellular proteins were harvested, and the expression levels of STAT1 were analysed quantitatively using Western blotting.

### 3.9 RNA‐Seq

GC1 cells were transfected with si‐NC, si‐*Senp8* or si‐*Stat1* and total RNA was extracted from each group after transfection. Three independent biological replicates were prepared for each experimental condition. RNA integrity and purity were assessed before library construction, and only RNA samples that met the quality control requirements for transcriptome sequencing were used for downstream analysis. Polyadenylated mRNA was enriched using oligo (dT)‐conjugated magnetic beads and subsequently fragmented in a divalent cation‐containing buffer under controlled conditions. First‐strand and second‐strand cDNA synthesis, end repair, adapter ligation and PCR amplification were performed according to the standard NEB library preparation protocol. The libraries were initially quantified using a Qubit 2.0 Fluorometer, and insert size distribution was examined using an Agilent 2100 Bioanalyzer. After library quality assessment, the effective concentration of each library was determined by qRT‐PCR. Qualified libraries were pooled in equimolar amounts according to their effective concentrations and the required sequencing depth, followed by Illumina high‐throughput sequencing.

For bioinformatic analysis, raw sequencing reads were first subjected to quality control to remove adapter sequences and low‐quality reads, thereby generating clean reads for downstream analysis. Clean reads were aligned to the mouse reference genome, and gene‐level read counts were generated for each sample. Differential expression analysis was performed by comparing si‐*Senp8* with si‐NC and si‐*Stat1* with si‐NC, respectively, using three biological replicates per group. *p* values obtained from the differential expression analysis were corrected for multiple testing using the Benjamini‐Hochberg false discovery rate (FDR) method. Genes were defined as DEGs when they met all of the following criteria: an absolute log2 fold change (|log2FC|) > 0.65, FDR‐adjusted *p* value (*q* value) < 0.05, and consistent expression changes among biological replicates. Upregulated and downregulated DEGs were subsequently used for volcano plot visualisation, heatmap clustering and overlap analysis between the si‐*Senp8* and si‐*Stat1* datasets. The overlapping DEGs identified from the two comparisons were considered candidate shared downstream targets regulated by SENP8 and STAT1 and were used to guide subsequent mechanistic validation.

### 3.10 Molecular Interaction Prediction

The structural model of Stat1 (P42225) was downloaded from the AlphaFold database (AF‐P42225‐F1) [22]. The potential DNA elements around the TSS were predicted by using the Tfinder tool based on the validated motif of human STAT1 (JASPAR ID: MA0137.2) [23, 24]. The flanking DNA sequences (−2 to 2 kb) of *Ccl2* and *Ccl5* around the TSS sites were based on the GRCm39 mouse genome. The top scored DNA element for *Ccl2* or *Ccl5* was docked to the DNA binding region (STAT1_DBD: 317–564) of Stat1 protein by using the local version of HDOCK [24].

### 3.11 Luciferase Reporter Assay

The empty vector, *Ccl2* and *Ccl5* promoters, as well as promoter mutants, were subcloned into the pGL6 luciferase vector. Subsequently, pGL6‐EV, pGL6‐*Ccl2*‐promoter‐WT, pGL6‐Ccl2‐promoter‐MUT, pGL6‐Ccl5‐promoter‐WT and pGL6‐*Ccl5*‐promoter‐MUT were successfully constructed. Next, the relevant plasmids were transfected into GC1 cells. The Renilla luciferase vector served as an internal control reporter gene. Finally, the luciferase activity was assayed using the luciferase reporter gene assay system.

### 3.12 ChIP‐qPCR

As previously reported, ChIP was carried out using the EZ‐ChIP kit (Millipore, Billerica, MA, USA) [25]. Specifically, cells were harvested and cross‐linked with 1% formaldehyde. Subsequently, the chromatin was fragmented into approximately 500‐base‐pair segments by employing a Branson Sonicator 250. Thereafter, the DNA‐protein complexes were incubated with specific antibodies. Meanwhile, approximately 10% of the initial sample was reserved as the input control. Eventually, the immunoprecipitated DNA was analysed via real‐time quantitative polymerase chain reaction (real‐time qPCR).

### 3.13 Statistical Analysis

Data analysis was performed with GraphPad Prism 10. Each experiment was independently repeated a minimum of three times to ensure the reliability of the number of biological replicates. Raw data collected through standardized data acquisition methods were processed using specific quantification procedures, wherein data are presented as mean values accompanied by standard deviation (±SD) to reflect central tendency and dispersion. For statistical comparisons, Student's *t*‐test and one‐way ANOVA were utilized. Statistical significance was defined as a *p* value below 0.05.

## Author Contributions

**Meng Liu:** investigation, methodology, writing. **Xiya Qiu:** methodology. **Wenying Qu:** methodology. **Mingyuan Bao:** methodology. **Yuxuan Feng:** methodology. **Xiaochu Wang:** methodology. **Yue Ma:** bioinformatics analysis. **Tao Zhou:** bioinformatics analysis. **Bo Zheng:** conceptualisation, funding acquisition, project administration, writing, supervision. **Yu Xia:** conceptualisation, writing, supervision. **Shunyu Hou:** conceptualisation, project administration, supervision. **Qingxia Meng:** conceptualisation, project administration, supervision.

## Funding

This work was supported by the National Key R&D Program of China (2024YFC2706702), the National Natural Science Foundation of China (82271633), the Gusu Health Talent Research Funding (GSWS2023012) and the Suzhou Science and Technology Bureau Project (SYWD2024007).

## Conflicts of Interest

The authors declare no conflicts of interest.

## Supporting information


**Figure S1:** SENP8 regulates STAT1 stability and activation in GC‐1 cells. (A) The diagram identifying proteins that interact with SENP8 in GC1. Flag‐SENP8 plasmid and empty vector was transfected into GC1. Total protein was harvested and subjected to immunoprecipitation (IP), with anti‐Flag beads, SDS‐PAGE of the immunoprecipitated products, in‐gel digestion and LC–MS/MS (By Figdraw). (B) Schematic diagram of STAT1 and domain mutations. (C) *Stat1* mRNA level in GC1 cells transfected with si‐NC and si‐*Stat1*, *n* = 3 per group. (D) STAT1 levels in GC1 cells under *Stat1* knockdown. (E) Statistical chart of D. *n* = 3 per group. (F) Cell Counting Kit‐8 (CCK8) assays showed the proliferation of GC1 after *Stat1* knockdown, *n* = 6 per group. (G and I) Colony formation assays were performed to assess the proliferative ability of *Stat1*‐knockdown GC1, *n* = 3 per group. (H and J) Transwell experiments showed that the migration of GC1 was reduced after *Stat1* knockdown, *n* = 3 per group. Scale bar = 100 μm. (K and L) STAT1, p‐STAT1(Tyr701) and p‐STAT1(Ser727) protein expression in GC1 after *Senp8* knockdown (si‐*Senp8* vs. si‐NC), *n* = 3 per group. (M and N) STAT1, p‐STAT1(Tyr701) and p‐STAT1(Ser727) expression in GC1 following SENP8 overexpression, *n* = 3 per group. Each experiment was independently repeated three times. The relevant *p* values had been marked in the figures.
**Figure S2:** SENP8 maintains STAT1 stability and supports proliferation and migration in human SSCs. (A) *SENP8* mRNA level in spermatogonial stem cells (SSCs) transfected with control siRNA (si‐NC) or *SENP8*‐targeting siRNA (si‐*SENP8*), *n* = 3 per group. (B and C) STAT1 protein expression in SSCs after *SENP8* knockdown, *n* = 3 per group. (D) CCK8 assay of SSCs proliferation after *SENP8* knockdown, *n* = 6 per group. (E and F) Transwell experiments showing reduced migration in *SENP8*‐knockdown SSCs, *n* = 3 per group. Scale bar = 100 μm. (G) STAT1 neddylation in SSCs after *SENP8* knockdown (si‐*SENP8*) versus control (si‐NC), detected by anti‐NEDD8 immunoblotting (IB) of STAT1 immunoprecipitates (IPs) from MG132 (20 μM, 6 h)‐treated cells. Each experiment was independently repeated three times. The relevant *p* values had been marked in the figures.
**Figure S3:** SENP8 regulates TM3 cell proliferation and migration independently of STAT1 stability. (A) *Senp8* mRNA level in TM3 cells transfected with control siRNA (si‐NC) or *Senp8*‐targeting siRNA (si‐*Senp8*), *n* = 3 per group. (B and C) STAT1 protein expression in TM3 cells after *Senp8* knockdown, *n* = 3 per group. (D) CCK8 assay of TM3 proliferation after *Senp8* knockdown, *n* = 6 per group. (E and G) Colony formation assays were performed to assess the proliferative ability of *Senp8*‐knockdown TM3 cells, *n* = 3 per group. (F and H) Transwell experiments showing reduced migration in *Senp8*‐knockdown TM3 cells, *n* = 3 per group. Scale bar = 100 μm. Each experiment was independently repeated three times. The relevant *p* values had been marked in the figures.
**Figure S4:** SENP8 maintains STAT1 stability through K240 de‐neddylation and a noncanonical degradation mechanism. (A) si‐NC or si‐*Senp8*, Myc‐NEDD8 plasmid and pEGFP‐STAT1 plasmid were co‐transfected into GC1 cells. These cells were followed by incubation with or without MG132 (20 μM) for 6 h. Then GFP‐beads were used for co‐immunoprecipitation (co‐IP). The level of STAT1 ubiquitination‐like modification was observed using Myc antibody. (B) EV or Flag‐SENP8, Myc‐NEDD8 plasmid and pEGFP‐STAT1 plasmid were co‐transfected into GC1 cells, ± MG132 (20 μM, 6 h). Then GFP‐beads were used for co‐immunoprecipitation (co‐IP). The level of STAT1 ubiquitination‐like modification was observed using Myc antibody. (C) STAT1 neddylation in GC1 cells after *Senp8* knockdown (si‐*Senp8*) versus control (si‐NC), detected by anti‐NEDD8 immunoblotting (IB) of STAT1 immunoprecipitates (IPs) from Epoxomicin (2 μM, 6 h)‐treated cells. (D) STAT1 neddylation in GC1 cells overexpressing SENP8 (Flag‐SENP8), detected as in C. (E) Co‐transfected STAT1‐GFP (WT, K240R, K379R, K592R, K636R, K637R, K652R, K673R, K685R), Myc‐NEDD8 plasmids and si‐*Senp8* or si‐NC in GC1 cells were subjected to Co‐IP assay, treated with MG132 (20 μM, 6 h). The level of STAT1 ubiquitination‐like modification was observed using Myc antibody. (F) CCK8 assay of GC1 proliferation after si‐NC + EV, si‐*Senp8* + EV and si‐*Senp8* + STAT1‐K240R‐GFP. *n* = 6 per group. (G and H) Transwell experiments showing corresponding changes in migration capacity in si‐NC + EV, si‐*Senp8* + EV and si‐*Senp8* + STAT1‐K240R‐GFP in GC1 cells, *n* = 3 per group. Scale bar = 100 μm. (I) STAT1, P27 and NRF2 protein levels in GC1 cells under the following conditions: si‐NC, si‐*Senp8*, si‐*Senp8* + MLN4924. (J) Statistical chart of I. *n* = 3 per group. (K) After *Senp8* knockdown (si‐*Senp8*) versus control (si‐NC) in GC1 cells, detected by anti‐UB immunoblotting (IB) of STAT1 immunoprecipitates (IPs) from MG132 (20 μM, 6 h)‐treated cells. Each experiment was independently repeated three times. The relevant *p* values had been marked in the figures.
**Figure S5:** SENP8 and STAT1 co‐regulate transcriptional programmes and chemokine‐related responses in GC‐1 cells. (A) Flowchart of RNA‐Seq for GC1 cells: Total RNA was isolated from GC1 cells transfected with si‐NC, si‐*Senp8* and si‐*Stat1*, followed by reverse transcription into complementary DNA (cDNA). Sequencing libraries were subsequently constructed and subjected to high‐throughput sequencing. Finally, bioinformatics analyses were performed using the raw sequencing data. (B) Volcano plot illustrating differentially expressed genes in GC1 cells transfected with si‐*Senp8*, generated based on RNA sequencing data. (C) Volcano plot illustrating differentially expressed genes in GC1 cells transfected with si‐*Stat1*, generated based on RNA sequencing data. (D) This bar chart illustrating the differentially expressed genes (DEGs) identified upon siRNA‐mediated knockdown of *Stat1* and *Senp8* (compared to the nontargeting control, si‐NC) as well as the intersection analysis of these two DEG sets to identify shared regulatory targets. (E and F) Heatmap respectively illustrating 40 overlapping differentially expressed genes (DEGs) in *Senp8* knockdown (compared to the nontargeting control, si‐NC), *Stat1* knockdown (compared to the nontargeting control, si‐NC).
**Figure S6:** CCL2 and CCL5 support germ cell proliferation and migration. (A) *Ccl2* mRNA levels in GC1 transfected with si‐NC and si‐*Ccl2*, *n* = 3 per group. (B) CCL2 protein levels in GC1 cells under *Ccl2* knockdown. (C) Statistical chart of B. *n* = 3 per group. (D) Proliferation of GC1 cells following *Ccl2* knockdown assessed by CCK‐8 assay, *n* = 6 per group. (E and G) Proliferative ability of *Ccl2*‐knockdown GC1 cells analysed by colony formation assay, *n* = 3 per group. (F and H) Migration of *Ccl2*‐knockdown GC1 cells analysed by Transwell assay, *n* = 3 per group. Scale bar = 100 μm. (I) *Ccl5* mRNA levels in GC1 transfected with si‐NC and si‐*Ccl5*, *n* = 3 per group. (J) CCL5 protein levels in GC1 cells under *Ccl5* knockdown. (K) Statistical chart of J. *n* = 3 per group. (L) Proliferation of GC1 cells following *Ccl5* knockdown assessed by CCK‐8 assay, *n* = 6 per group. (M and O) Proliferative ability of *Ccl5*‐knockdown GC1 cells analysed by colony formation assay, *n* = 3 per group. (N and P) Migration of *Ccl5*‐knockdown GC1 cells analysed by Transwell assay, *n* = 3 per group. Scale bar = 100 μm. Data represent mean ± SD from three independent experiments. The relevant *p* values had been marked in the figures.
**Figure S7:** STAT1 directly activates *Ccl2* and *Ccl5* transcription through promoter binding. (A) Distribution of the relative positions and scores of binding sites among *Ccl2* and *Ccl5* promoters (−2 to 2 kb regions of TSS). (B) Docking and confidence scores for the best models (with most residues located in the STAT1_DBD domain) of STAT1‐*Ccl2* and STAT1‐*Ccl5* complexes respectively. (C) Interacting links between STAT1 protein and the binding DNA elements (15 bases) of *Ccl2* and *Ccl5*. (D) Comparison of the common receptor residues between STAT1‐*Ccl2* and STAT1‐*Ccl5*. (E) The changes in relative *Ccl2* expression after treatment with different concentrations of STAT1‐WT‐GFP and STAT1‐MUT‐GFP. (F) The changes in relative *Ccl5* expression after treatment with different concentrations of STAT1‐WT‐GFP and STAT1‐MUT‐GFP. (G) After treating GC1 cells with GFP, STAT1‐WT‐GFP and STAT1‐MUT‐GFP for 48 h, dual‐luciferase reporter assays were performed to validate the luciferase activities of the empty vector (EV), *Ccl2*‐promoter‐WT and *Ccl2*‐promoter‐MUT reporter constructs. (H) After treating GC1 cells with GFP, STAT1‐WT‐GFP and STAT1‐MUT‐GFP for 48 h, dual‐luciferase reporter assays were performed to validate the luciferase activities of the EV, *Ccl5*‐promoter‐WT and *Ccl5*‐promoter‐MUT reporter constructs. For E‐H, *n* = 3 per group. Each experiment was independently repeated three times. The relevant *p* values had been marked in the figures.
**Figure S8:** Identification of Key STAT1 Binding Regions Required for Ccl2 and Ccl5 Promoter Activation. (A) *Ccl2* and *Ccl5* shared six common binding sites in the STAT1‐DBR region. Deletion mutants were generated for each individual site. After treating GC1 cells with GFP, STAT1‐WT‐GFP and the respective deletion mutants for 48 h, dual‐luciferase reporter assays were performed to validate the luciferase activities of the empty vector (EV), *Ccl2*‐promoter‐WT and *Ccl2*‐promoter‐MUT reporter constructs. (B) After treating GC1 cells with GFP, STAT1‐WT‐GFP and the respective deletion mutants for 48 h, dual‐luciferase reporter assays were performed to validate the luciferase activities of the EV, *Ccl5*‐promoter‐WT and *Ccl5*‐promoter‐MUT reporter constructs. For A‐B, *n* = 3 per group. The relevant *p* values had been marked in the figures.
**Figure S9:** CCL5, but Not CCL2, maintains STAT1 phosphorylation in the SENP8–STAT1 axis. (A and B) Changes in protein levels of STAT1, p‐STAT1 (Tyr701) and p‐STAT1 (Ser727) following *Ccl2* knockdown, *n* = 3 per group. (C and D) Changes in protein levels of STAT1, p‐STAT1 (Tyr701) and p‐STAT1 (Ser727) following *Ccl5* knockdown, *n* = 3 per group. (E) Proposed mechanism by which SENP8 regulates GC1 cell proliferation and migration. Each experiment was independently repeated three times. The relevant *p* values had been marked in the figures.

## Data Availability

The mass spectrometry proteomics data have been deposited to the ProteomeXchange Consortium via the PRIDE repository with the dataset identifier PXD071924. The transcriptome sequencing data have been deposited in ArrayExpress under accession E‐MTAB‐16423.
