# Supplementary material for: A Noncanonical Protein Degradation Pathway That Regulates Germ Cell Maintenance
Source: Cell Prolif. 2026 Jul 14;59(10):e70259. doi: 10.1111/cpr.70259 (PMC13370090; doi:10.1111/cpr.70259)
Supplement: Supplementary file 1 [file CPR-59-e70259-s001.docx]

**Supplementary Figure 1** SENP8 Regulates STAT1 Stability and Activation in GC-1 Cells. (A) The diagram identifying proteins that interact with SENP8 in GC1. Flag-SENP8 plasmid and empty vector was transfected into GC1. Total protein was harvested and subjected to immunoprecipitation (IP), with anti-Flag beads, SDS-PAGE of the immunoprecipitated products, in-gel digestion, and LC-MS/MS (By Figdraw). (B) Schematic diagram of STAT1 and domain mutations. (C) *Stat1* mRNA level in GC1 cells transfected with si-NC and si-*Stat1*, n = 3 per group. (D) STAT1 levels in GC1 cells under *Stat1* knockdown. (E) Statistical chart of D. n = 3 per group. (F) Cell Counting Kit-8 (CCK8) assays showed the proliferation of GC1 after *Stat1* knockdown, n = 6 per group. (G, I) Colony formation assays were performed to assess the proliferative ability of *Stat1*-knockdown GC1, n = 3 per group. (H, J) Transwell experiments showed that the migration of GC1 was reduced after *Stat1* knockdown, n = 3 per group. Scale bar = 100 μm. (K,L) STAT1, p-STAT1(Tyr701) and p-STAT1(Ser727) protein expression in GC1 after *Senp8* knockdown (si-*Senp8* vs. si-NC), n = 3 per group. (M,N) STAT1, p-STAT1(Tyr701), and p-STAT1(Ser727) expression in GC1 following SENP8 overexpression, n = 3 per group. Each experiment was independently repeated three times. The relevant p-values had been marked in the figures.

**Supplementary Figure 2** SENP8 Maintains STAT1 Stability and Supports Proliferation and Migration in Human SSCs. (A) *SENP8* mRNA level in Spermatogonial Stem Cells (SSCs) transfected with control siRNA (si-NC) or *SENP8*-targeting siRNA (si-*SENP8*), n = 3 per group. (B,C) STAT1 protein expression in SSCs after *SENP8* knockdown, n = 3 per group. (D) CCK8 assay of SSCs proliferation after *SENP8* knockdown, n = 6 per group. (E,F) Transwell experiments showing reduced migration in *SENP8*-knockdown SSCs, n = 3 per group. Scale bar = 100 μm. (G) STAT1 neddylation in SSCs after *SENP8* knockdown (si-*SENP8*) versus control (si-NC), detected by anti-NEDD8 immunoblotting (IB) of STAT1 immunoprecipitates (IPs) from MG132 (20μM, 6h)-treated cells. Each experiment was independently repeated three times. The relevant p-values had been marked in the figures.

**Supplementary Figure 3** SENP8 Regulates TM3 Cell Proliferation and Migration Independently of STAT1 Stability. (A) *Senp8* mRNA level in TM3 cells transfected with control siRNA (si-NC) or *Senp8*-targeting siRNA (si-*Senp8*), n = 3 per group. (B,C) STAT1 protein expression in TM3 cells after *Senp8* knockdown, n = 3 per group. (D) CCK8 assay of TM3 proliferation after *Senp8* knockdown, n = 6 per group. (E,G) Colony formation assays were performed to assess the proliferative ability of *Senp8*-knockdown TM3 cells, n = 3 per group. (F,H) Transwell experiments showing reduced migration in *Senp8*-knockdown TM3 cells, n = 3 per group. Scale bar = 100 μm. Each experiment was independently repeated three times. The relevant p-values had been marked in the figures.

**Supplementary Figure 4** SENP8 Maintains STAT1 Stability Through K240 De-Neddylation and a Non-Canonical Degradation Mechanism. (A) si-NC or si-*Senp8*, Myc-NEDD8 plasmid and pEGFP-STAT1 plasmid were co-transfected into GC1 cells. These cells were followed by incubation with or without MG132 (20μM) for six hours. Then GFP-beads were used for co-immunoprecipitation (co-IP). The level of STAT1 ubiquitination-like modification was observed using Myc antibody. (B) EV or Flag-SENP8, Myc-NEDD8 plasmid and pEGFP-STAT1 plasmid were co-transfected into GC1 cells, ±MG132 (20μM, 6h). Then GFP-beads were used for co-immunoprecipitation (co-IP). The level of STAT1 ubiquitination-like modification was observed using Myc antibody. (C) STAT1 neddylation in GC1 cells after *Senp8* knockdown (si-*Senp8*) versus control (si-NC), detected by anti-NEDD8 immunoblotting (IB) of STAT1 immunoprecipitates (IPs) from Epoxomicin (2μM, 6h)-treated cells. (D) STAT1 neddylation in GC1 cells overexpressing SENP8 (Flag-SENP8), detected as in C. (E) Co-transfected STAT1-GFP (WT, K240R, K379R, K592R, K636R, K637R, K652R, K673R, K685R), Myc-NEDD8 plasmids and si-*Senp8* or si-NC in GC1 cells were subjected to Co-IP assay, treated with MG132 (20μM, 6h). The level of STAT1 ubiquitination-like modification was observed using Myc antibody. (F) CCK8 assay of GC1 proliferation after si-NC+EV, si-*Senp8*+EV and si-*Senp8*+STAT1-K240R-GFP. n = 6 per group. (G,H) Transwell experiments showing corresponding changes in migration capacity in si-NC+EV, si-*Senp8*+EV and si-*Senp8*+STAT1-K240R-GFP in GC1 cells, n = 3 per group. Scale bar = 100 μm. (I) STAT1, P27 and NRF2 protein levels in GC1 cells under the following conditions: si-NC, si-*Senp8*, si-*Senp8*+MLN4924. (J) Statistical chart of I. n = 3 per group. (K) After *Senp8* knockdown (si-*Senp8*) versus control (si-NC) in GC1 cells, detected by anti-UB immunoblotting (IB) of STAT1 immunoprecipitates (IPs) from MG132 (20μM, 6h)-treated cells. Each experiment was independently repeated three times. The relevant p-values had been marked in the figures.

**Supplementary Figure 5** SENP8 and STAT1 Co-regulate Transcriptional Programs and Chemokine-Related Responses in GC-1 Cells. (A) Flowchart of RNA-Seq for GC1 cells: Total RNA was isolated from GC1 cells transfected with si-NC, si-*Senp8*, and si-*Stat1*, followed by reverse transcription into complementary DNA (cDNA). Sequencing libraries were subsequently constructed and subjected to high-throughput sequencing. Finally, bioinformatics analyses were performed using the raw sequencing data. (B) Volcano plot illustrating differentially expressed genes in GC1 cells transfected with si-*Senp8*, generated based on RNA sequencing data. (C) Volcano plot illustrating differentially expressed genes in GC1 cells transfected with si-*Stat1*, generated based on RNA sequencing data. (D) This bar chart illustrating the differentially expressed genes (DEGs) identified upon siRNA-mediated knockdown of *Stat1* and *Senp8* (compared to the non-targeting control, si-NC) as well as the intersection analysis of these two DEG sets to identify shared regulatory targets. (E,F) Heatmap respectively illustrating 40 overlapping differentially expressed genes (DEGs) in *Senp8* knockdown (compared to the non-targeting control, si-NC), *Stat1* knockdown (compared to the non-targeting control, si-NC).

**Supplementary Figure 6** CCL2 and CCL5 Support Germ Cell Proliferation and Migration. (A) *Ccl2* mRNA levels in GC1 transfected with si-NC and si-*Ccl2*, n = 3 per group. (B) CCL2 protein levels in GC1 cells under *Ccl2* knockdown. (C) Statistical chart of B. n = 3 per group. (D) Proliferation of GC1 cells following *Ccl2* knockdown assessed by CCK-8 assay, n = 6 per group. (E,G) Proliferative ability of *Ccl2*-knockdown GC1 cells analyzed by colony formation assay, n = 3 per group. (F,H) Migration of *Ccl2*-knockdown GC1 cells analyzed by Transwell assay, n = 3 per group. Scale bar = 100 μm. (I) *Ccl5* mRNA levels in GC1 transfected with si-NC and si-*Ccl5*, n = 3 per group. (J) CCL5 protein levels in GC1 cells under *Ccl5* knockdown. (K) Statistical chart of J. n = 3 per group. (L) Proliferation of GC1 cells following *Ccl5* knockdown assessed by CCK-8 assay, n = 6 per group. (M,O) Proliferative ability of *Ccl5*-knockdown GC1 cells analyzed by colony formation assay, n = 3 per group. (N,P) Migration of *Ccl5*-knockdown GC1 cells analyzed by Transwell assay, n = 3 per group. Scale bar = 100 μm. Data represent mean ± SD from three independent experiments. The relevant p-values had been marked in the figures.

**Supplementary Figure 7** STAT1 Directly Activates *Ccl2* and *Ccl5* Transcription Through Promoter Binding. (A) Distribution of the relative positions and scores of binding sites among *Ccl2* and *Ccl5* promoters (-2 kb to 2 kb regions of TSS). (B) Docking and confidence scores for the best models (with most residues located in the STAT1_DBD domain) of STAT1-*Ccl2* and STAT1-*Ccl5* complexes respectively. (C) Interacting links between STAT1 protein and the binding DNA elements (15 bases) of *Ccl2* and *Ccl5*. (D) Comparison of the common receptor residues between STAT1-*Ccl2* and STAT1-*Ccl5*. (E) The changes in relative *Ccl2* expression after treatment with different concentrations of STAT1-WT-GFP and STAT1-MUT-GFP. (F) The changes in relative *Ccl5* expression after treatment with different concentrations of STAT1-WT-GFP and STAT1-MUT-GFP. (G) After treating GC1 cells with GFP, STAT1-WT-GFP, and STAT1-MUT-GFP for 48 hours, dual-luciferase reporter assays were performed to validate the luciferase activities of the empty vector (EV), *Ccl2*-promoter-WT, and *Ccl2*-promoter-MUT reporter constructs. (H) After treating GC1 cells with GFP, STAT1-WT-GFP, and STAT1-MUT-GFP for 48 hours, dual-luciferase reporter assays were performed to validate the luciferase activities of the empty vector (EV), *Ccl5*-promoter-WT, and *Ccl5*-promoter-MUT reporter constructs. For E-H, n = 3 per group. Each experiment was independently repeated three times. The relevant p-values had been marked in the figures.

**Supplementary Figure 8** Identification of Key STAT1 Binding Regions Required for Ccl2 and Ccl5 Promoter Activation. (A) *Ccl2* and *Ccl5* shared six common binding sites in the STAT1-DBR region. Deletion mutants were generated for each individual site. After treating GC1 cells with GFP, STAT1-WT-GFP, and the respective deletion mutants for 48 hours, dual-luciferase reporter assays were performed to validate the luciferase activities of the empty vector (EV), *Ccl2*-promoter-WT, and *Ccl2*-promoter-MUT reporter constructs. (B) After treating GC1 cells with GFP, STAT1-WT-GFP, and the respective deletion mutants for 48 hours, dual-luciferase reporter assays were performed to validate the luciferase activities of the empty vector (EV), *Ccl5*-promoter-WT, and *Ccl5*-promoter-MUT reporter constructs. For A-B, n = 3 per group. The relevant p-values had been marked in the figures.

**Supplementary Figure 9** CCL5, but Not CCL2, Maintains STAT1 Phosphorylation in the SENP8–STAT1 Axis. (A,B) Changes in protein levels of STAT1, p-STAT1 (Tyr701), and p-STAT1 (Ser727) following *Ccl2* knockdown, n = 3 per group. (C,D) Changes in protein levels of STAT1, p-STAT1 (Tyr701), and p-STAT1 (Ser727) following *Ccl5* knockdown, n = 3 per group. (E) Proposed mechanism by which SENP8 regulates GC1 cell proliferation and migration. Each experiment was independently repeated three times. The relevant p-values had been marked in the figures.
