# Supplementary material for: A Noncanonical Protein Degradation Pathway That Regulates Germ Cell Maintenance
Source: Cell Prolif. 2026 Jul 14:e70259. Online ahead of print. doi: 10.1111/cpr.70259 (PMC13370090; doi:10.1111/cpr.70259)
Supplement: Supplementary file 1 — Figure S1: SENP8 regulates STAT1 stability and activation in GC‐1 cells. (A) The diagram identifying proteins that interact with SENP8 in GC1. Flag‐SENP8 plasmid and empty vector was transfected into GC1. Total protein was harvested and subjected to immunoprecipitation (IP), with anti‐Flag beads, SDS‐PAGE of the immunoprecipitated products, in‐gel digestion and LC–MS/MS (By Figdraw). (B) Schematic diagram of STAT1 and domain mutations. (C) Stat1 mRNA level in GC1 cells transfected with si‐NC and si‐Stat1, n = 3 per group. (D) STAT1 levels in GC1 cells under Stat1 knockdown. (E) Statistical chart of D. n = 3 per group. (F) Cell Counting Kit‐8 (CCK8) assays showed the proliferation of GC1 after Stat1 knockdown, n = 6 per group. (G and I) Colony formation assays were performed to assess the proliferative ability of Stat1‐knockdown GC1, n = 3 per group. (H and J) Transwell experiments showed that the migration of GC1 was reduced after Stat1 knockdown, n = 3 per group. Scale bar = 100 μm. (K and L) STAT1, p‐STAT1(Tyr701) and p‐STAT1(Ser727) protein expression in GC1 after Senp8 knockdown (si‐Senp8 vs. si‐NC), n = 3 per group. (M and N) STAT1, p‐STAT1(Tyr701) and p‐STAT1(Ser727) expression in GC1 following SENP8 overexpression, n = 3 per group. Each experiment was independently repeated three times. The relevant p values had been marked in the figures. Figure S2: SENP8 maintains STAT1 stability and supports proliferation and migration in human SSCs. (A) SENP8 mRNA level in spermatogonial stem cells (SSCs) transfected with control siRNA (si‐NC) or SENP8‐targeting siRNA (si‐SENP8), n = 3 per group. (B and C) STAT1 protein expression in SSCs after SENP8 knockdown, n = 3 per group. (D) CCK8 assay of SSCs proliferation after SENP8 knockdown, n = 6 per group. (E and F) Transwell experiments showing reduced migration in SENP8‐knockdown SSCs, n = 3 per group. Scale bar = 100 μm. (G) STAT1 neddylation in SSCs after SENP8 knockdown (si‐SENP8) versus control (si‐NC), detected by ant [file CPR-9999-e70259-s001.docx]

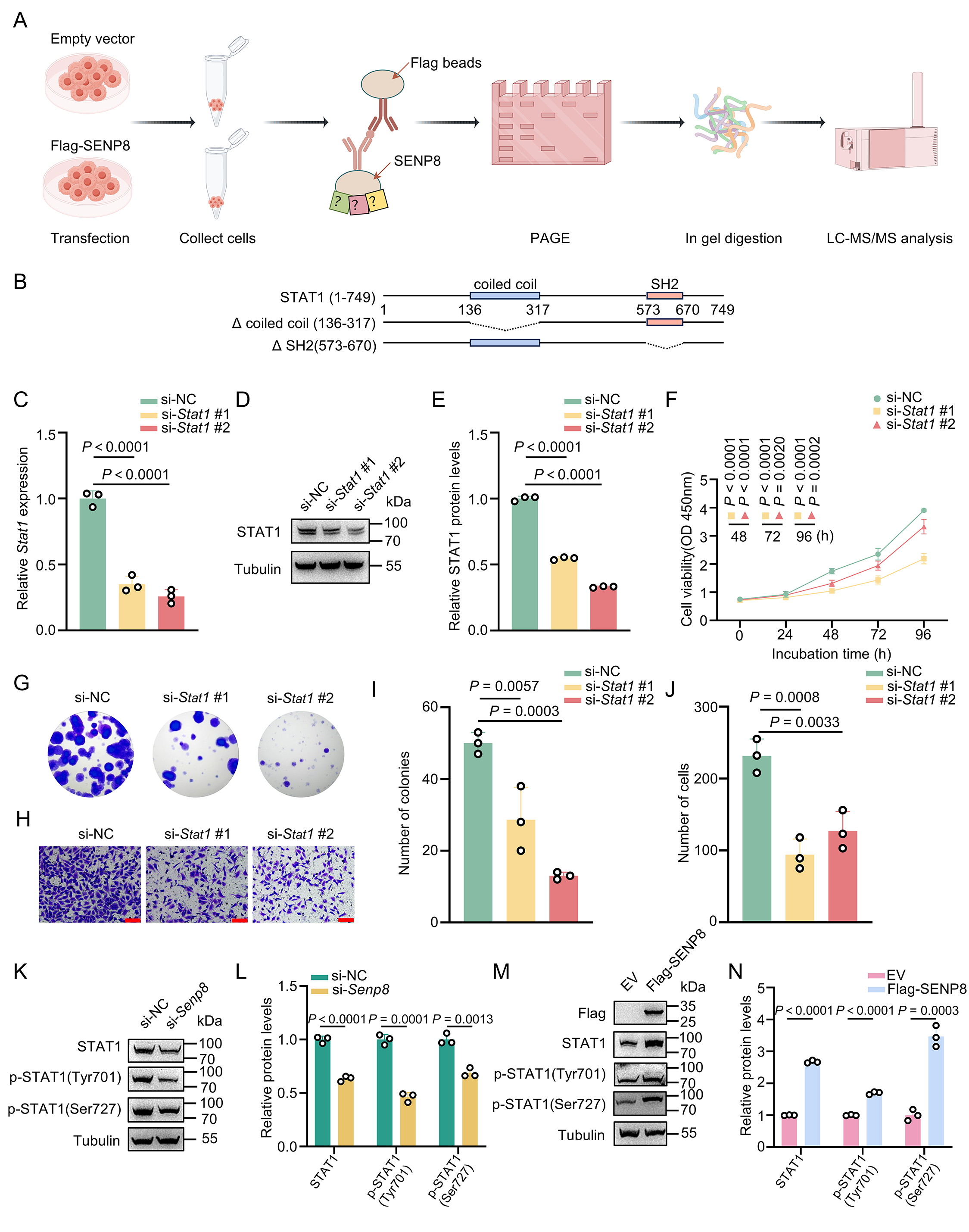


**Supplementary Figure 1** SENP8 Regulates STAT1 Stability and Activation in GC-1 Cells. (A) The diagram identifying proteins that interact with SENP8 in GC1. Flag-SENP8 plasmid and empty vector was transfected into GC1. Total protein was harvested and subjected to immunoprecipitation (IP), with anti-Flag beads, SDS-PAGE of the immunoprecipitated products, in-gel digestion, and LC-MS/MS (By Figdraw). (B) Schematic diagram of STAT1 and domain mutations. (C) *Stat1* mRNA level in GC1 cells transfected with si-NC and si-*Stat1*, n = 3 per group. (D) STAT1 levels in GC1 cells under *Stat1* knockdown. (E) Statistical chart of D. n = 3 per group. (F) Cell Counting Kit-8 (CCK8) assays showed the proliferation of GC1 after *Stat1* knockdown, n = 6 per group. (G, I) Colony formation assays were performed to assess the proliferative ability of *Stat1*-knockdown GC1, n = 3 per group. (H, J) Transwell experiments showed that the migration of GC1 was reduced after *Stat1* knockdown, n = 3 per group. Scale bar = 100 μm. (K,L) STAT1, p-STAT1(Tyr701) and p-STAT1(Ser727) protein expression in GC1 after *Senp8* knockdown (si-*Senp8* vs. si-NC), n = 3 per group. (M,N) STAT1, p-STAT1(Tyr701), and p-STAT1(Ser727) expression in GC1 following SENP8 overexpression, n = 3 per group. Each experiment was independently repeated three times. The relevant p-values had been marked in the figures.


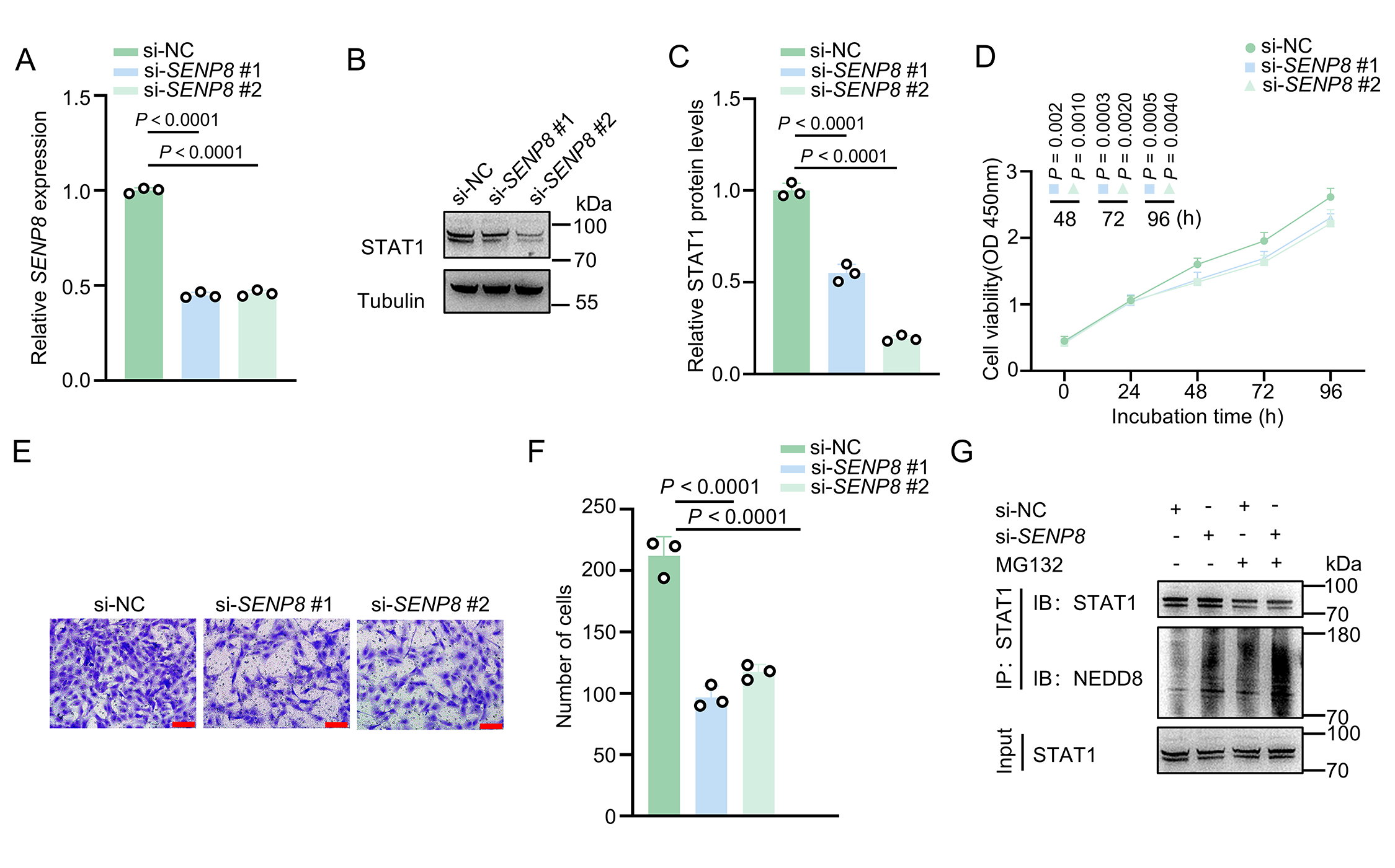


**Supplementary Figure 2** SENP8 Maintains STAT1 Stability and Supports Proliferation and Migration in Human SSCs. (A) *SENP8* mRNA level in Spermatogonial Stem Cells (SSCs) transfected with control siRNA (si-NC) or *SENP8*-targeting siRNA (si-*SENP8*), n = 3 per group. (B,C) STAT1 protein expression in SSCs after *SENP8* knockdown, n = 3 per group. (D) CCK8 assay of SSCs proliferation after *SENP8* knockdown, n = 6 per group. (E,F) Transwell experiments showing reduced migration in *SENP8*-knockdown SSCs, n = 3 per group. Scale bar = 100 μm. (G) STAT1 neddylation in SSCs after *SENP8* knockdown (si-*SENP8*) versus control (si-NC), detected by anti-NEDD8 immunoblotting (IB) of STAT1 immunoprecipitates (IPs) from MG132 (20μM, 6h)-treated cells. Each experiment was independently repeated three times. The relevant p-values had been marked in the figures.


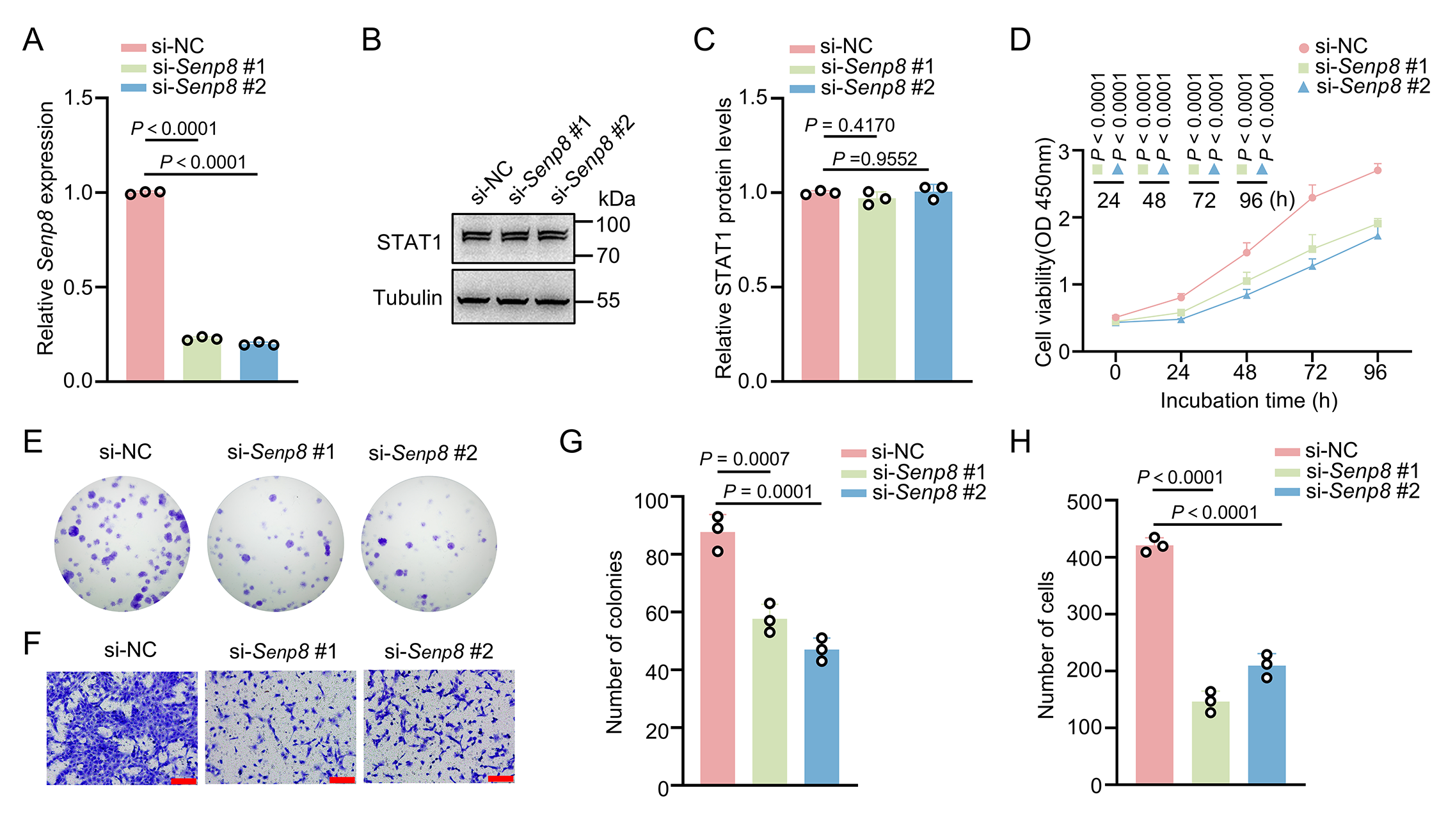


**Supplementary Figure 3** SENP8 Regulates TM3 Cell Proliferation and Migration Independently of STAT1 Stability. (A) *Senp8* mRNA level in TM3 cells transfected with control siRNA (si-NC) or *Senp8*-targeting siRNA (si-*Senp8*), n = 3 per group. (B,C) STAT1 protein expression in TM3 cells after *Senp8* knockdown, n = 3 per group. (D) CCK8 assay of TM3 proliferation after *Senp8* knockdown, n = 6 per group. (E,G) Colony formation assays were performed to assess the proliferative ability of *Senp8*-knockdown TM3 cells, n = 3 per group. (F,H) Transwell experiments showing reduced migration in *Senp8*-knockdown TM3 cells, n = 3 per group. Scale bar = 100 μm. Each experiment was independently repeated three times. The relevant p-values had been marked in the figures.


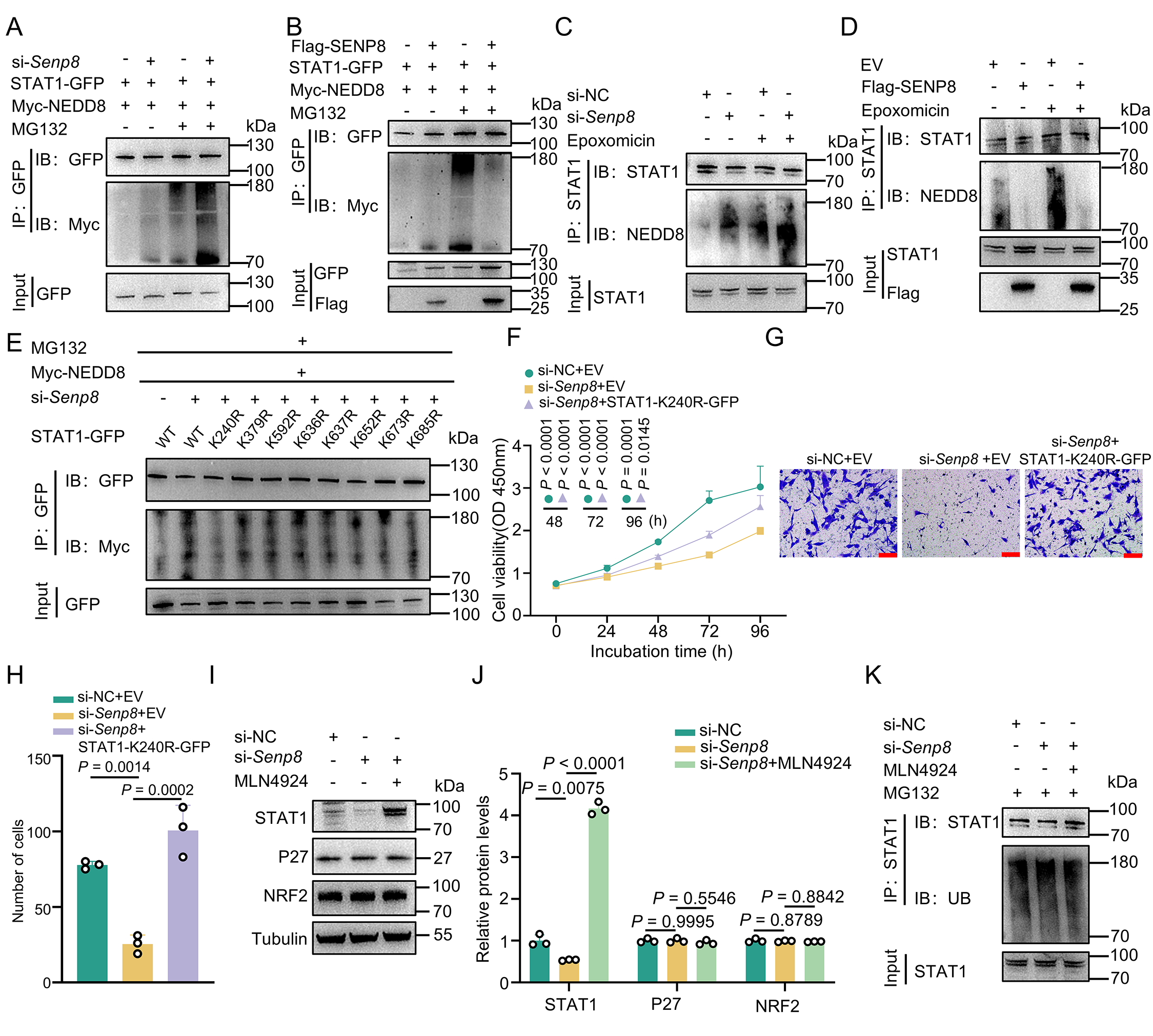


**Supplementary Figure 4** SENP8 Maintains STAT1 Stability Through K240 De-Neddylation and a Non-Canonical Degradation Mechanism. (A) si-NC or si-*Senp8*, Myc-NEDD8 plasmid and pEGFP-STAT1 plasmid were co-transfected into GC1 cells. These cells were followed by incubation with or without MG132 (20μM) for six hours. Then GFP-beads were used for co-immunoprecipitation (co-IP). The level of STAT1 ubiquitination-like modification was observed using Myc antibody. (B) EV or Flag-SENP8, Myc-NEDD8 plasmid and pEGFP-STAT1 plasmid were co-transfected into GC1 cells, ±MG132 (20μM, 6h). Then GFP-beads were used for co-immunoprecipitation (co-IP). The level of STAT1 ubiquitination-like modification was observed using Myc antibody. (C) STAT1 neddylation in GC1 cells after *Senp8* knockdown (si-*Senp8*) versus control (si-NC), detected by anti-NEDD8 immunoblotting (IB) of STAT1 immunoprecipitates (IPs) from Epoxomicin (2μM, 6h)-treated cells. (D) STAT1 neddylation in GC1 cells overexpressing SENP8 (Flag-SENP8), detected as in C. (E) Co-transfected STAT1-GFP (WT, K240R, K379R, K592R, K636R, K637R, K652R, K673R, K685R), Myc-NEDD8 plasmids and si-*Senp8* or si-NC in GC1 cells were subjected to Co-IP assay, treated with MG132 (20μM, 6h). The level of STAT1 ubiquitination-like modification was observed using Myc antibody. (F) CCK8 assay of GC1 proliferation after si-NC+EV, si-*Senp8*+EV and si-*Senp8*+STAT1-K240R-GFP. n = 6 per group. (G,H) Transwell experiments showing corresponding changes in migration capacity in si-NC+EV, si-*Senp8*+EV and si-*Senp8*+STAT1-K240R-GFP in GC1 cells, n = 3 per group. Scale bar = 100 μm. (I) STAT1, P27 and NRF2 protein levels in GC1 cells under the following conditions: si-NC, si-*Senp8*, si-*Senp8*+MLN4924. (J) Statistical chart of I. n = 3 per group. (K) After *Senp8* knockdown (si-*Senp8*) versus control (si-NC) in GC1 cells, detected by anti-UB immunoblotting (IB) of STAT1 immunoprecipitates (IPs) from MG132 (20μM, 6h)-treated cells. Each experiment was independently repeated three times. The relevant p-values had been marked in the figures.


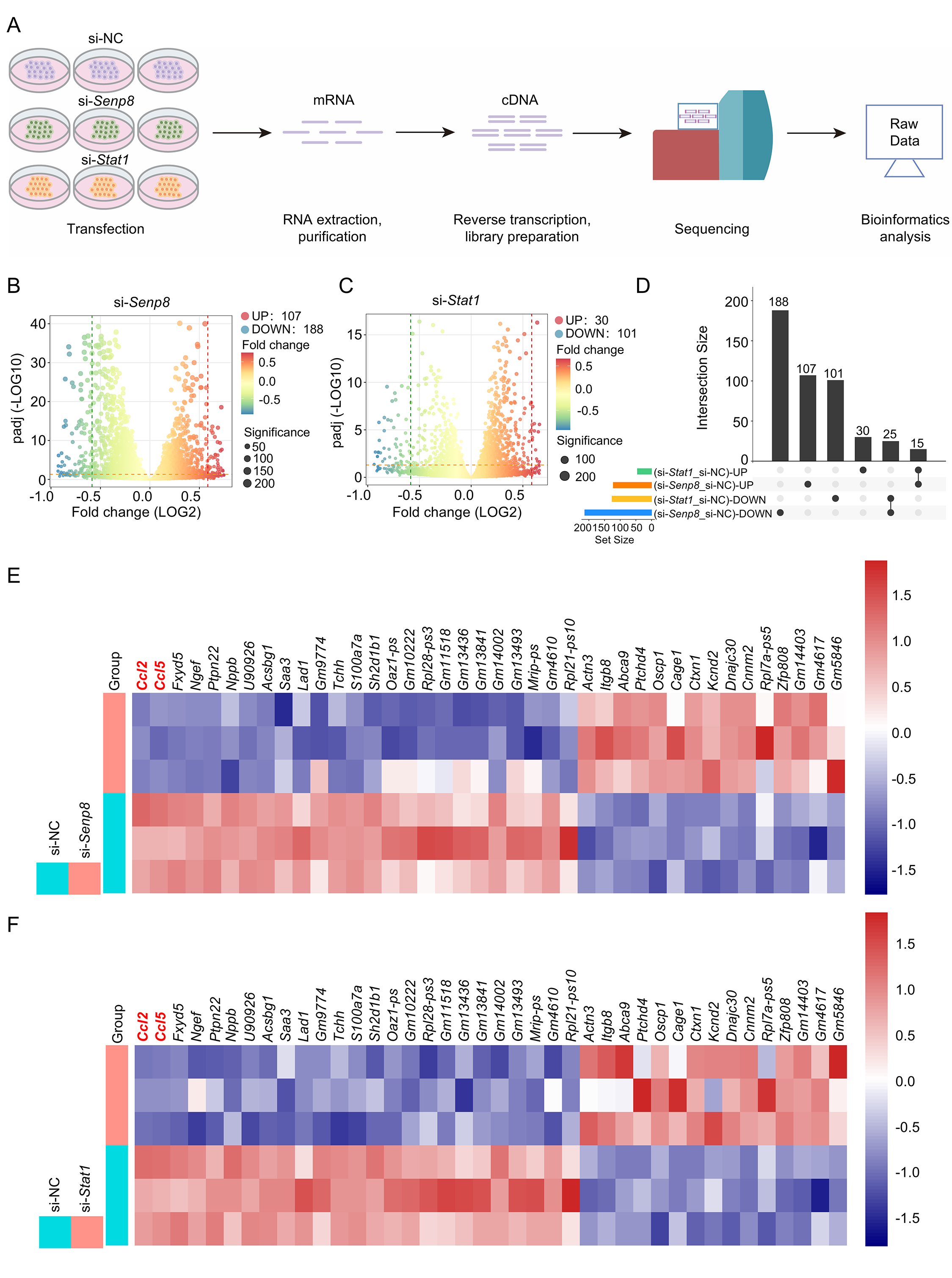


**Supplementary Figure 5** SENP8 and STAT1 Co-regulate Transcriptional Programs and Chemokine-Related Responses in GC-1 Cells. (A) Flowchart of RNA-Seq for GC1 cells: Total RNA was isolated from GC1 cells transfected with si-NC, si-*Senp8*, and si-*Stat1*, followed by reverse transcription into complementary DNA (cDNA). Sequencing libraries were subsequently constructed and subjected to high-throughput sequencing. Finally, bioinformatics analyses were performed using the raw sequencing data. (B) Volcano plot illustrating differentially expressed genes in GC1 cells transfected with si-*Senp8*, generated based on RNA sequencing data. (C) Volcano plot illustrating differentially expressed genes in GC1 cells transfected with si-*Stat1*, generated based on RNA sequencing data. (D) This bar chart illustrating the differentially expressed genes (DEGs) identified upon siRNA-mediated knockdown of *Stat1* and *Senp8* (compared to the non-targeting control, si-NC) as well as the intersection analysis of these two DEG sets to identify shared regulatory targets. (E,F) Heatmap respectively illustrating 40 overlapping differentially expressed genes (DEGs) in *Senp8* knockdown (compared to the non-targeting control, si-NC), *Stat1* knockdown (compared to the non-targeting control, si-NC).


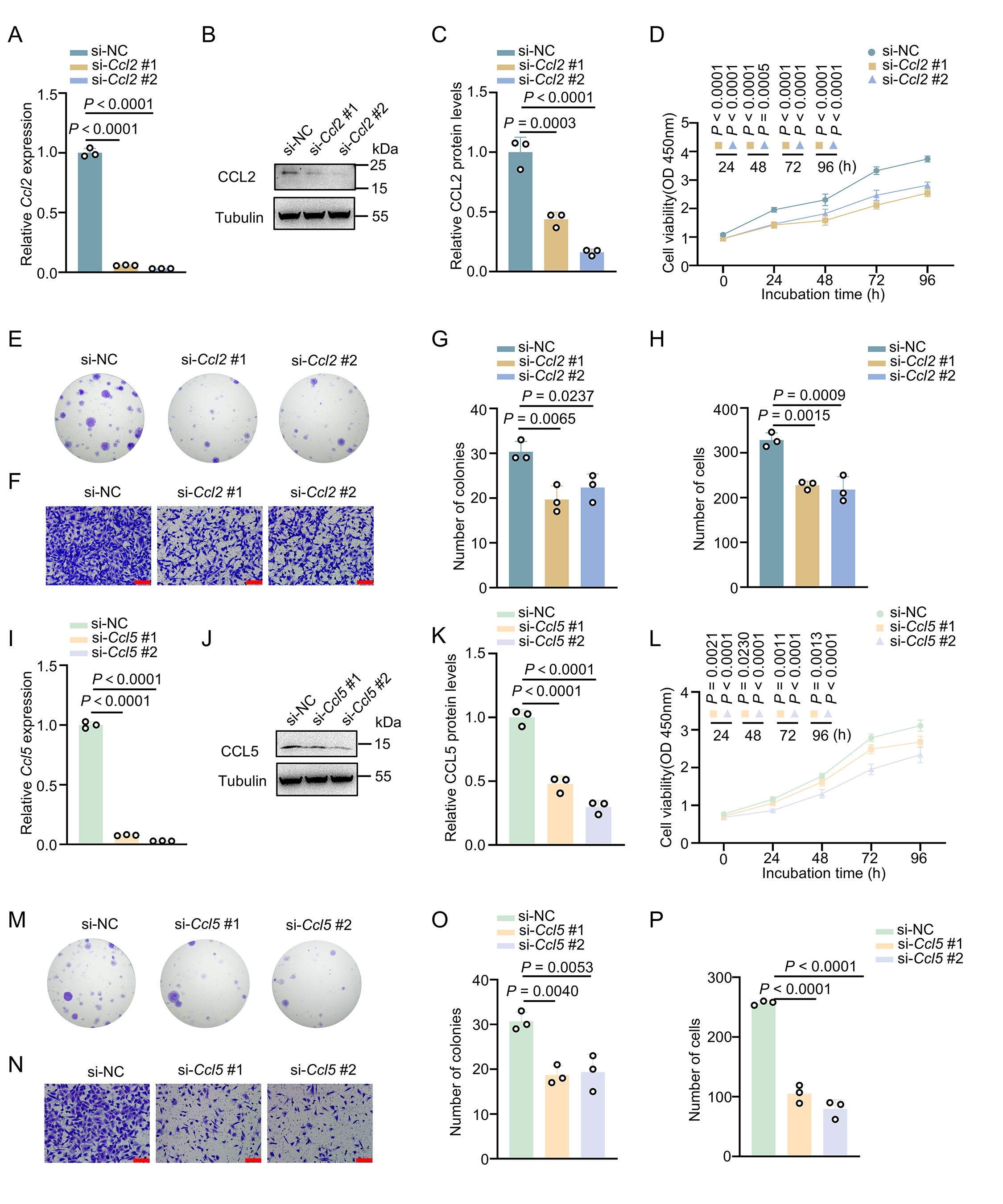


**Supplementary Figure 6** CCL2 and CCL5 Support Germ Cell Proliferation and Migration. (A) *Ccl2* mRNA levels in GC1 transfected with si-NC and si-*Ccl2*, n = 3 per group. (B) CCL2 protein levels in GC1 cells under *Ccl2* knockdown. (C) Statistical chart of B. n = 3 per group. (D) Proliferation of GC1 cells following *Ccl2* knockdown assessed by CCK-8 assay, n = 6 per group. (E,G) Proliferative ability of *Ccl2*-knockdown GC1 cells analyzed by colony formation assay, n = 3 per group. (F,H) Migration of *Ccl2*-knockdown GC1 cells analyzed by Transwell assay, n = 3 per group. Scale bar = 100 μm. (I) *Ccl5* mRNA levels in GC1 transfected with si-NC and si-*Ccl5*, n = 3 per group. (J) CCL5 protein levels in GC1 cells under *Ccl5* knockdown. (K) Statistical chart of J. n = 3 per group. (L) Proliferation of GC1 cells following *Ccl5* knockdown assessed by CCK-8 assay, n = 6 per group. (M,O) Proliferative ability of *Ccl5*-knockdown GC1 cells analyzed by colony formation assay, n = 3 per group. (N,P) Migration of *Ccl5*-knockdown GC1 cells analyzed by Transwell assay, n = 3 per group. Scale bar = 100 μm. Data represent mean ± SD from three independent experiments. The relevant p-values had been marked in the figures.


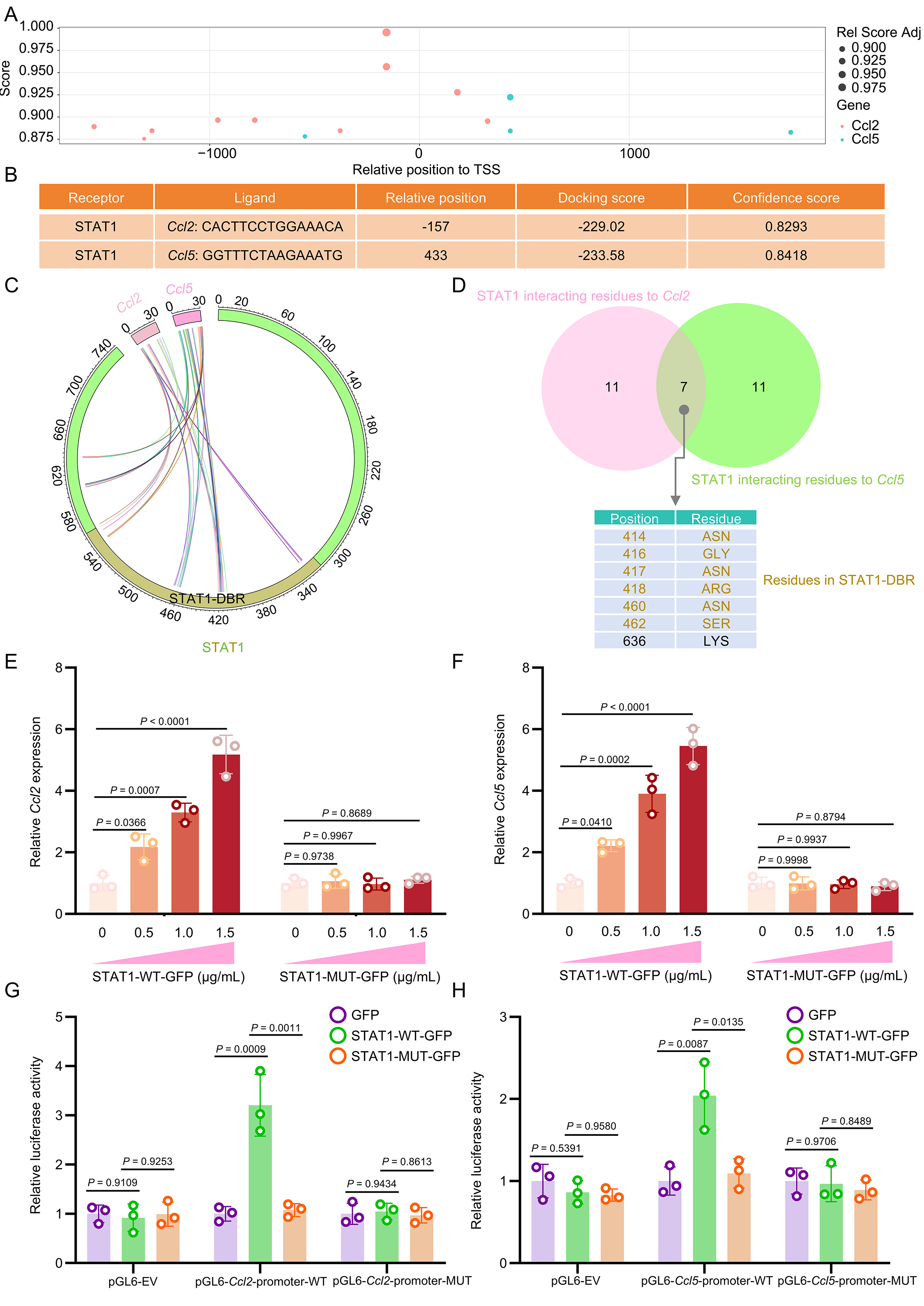


**Supplementary Figure 7** STAT1 Directly Activates *Ccl2* and *Ccl5* Transcription Through Promoter Binding. (A) Distribution of the relative positions and scores of binding sites among *Ccl2* and *Ccl5* promoters (-2 kb to 2 kb regions of TSS). (B) Docking and confidence scores for the best models (with most residues located in the STAT1_DBD domain) of STAT1-*Ccl2* and STAT1-*Ccl5* complexes respectively. (C) Interacting links between STAT1 protein and the binding DNA elements (15 bases) of *Ccl2* and *Ccl5*. (D) Comparison of the common receptor residues between STAT1-*Ccl2* and STAT1-*Ccl5*. (E) The changes in relative *Ccl2* expression after treatment with different concentrations of STAT1-WT-GFP and STAT1-MUT-GFP. (F) The changes in relative *Ccl5* expression after treatment with different concentrations of STAT1-WT-GFP and STAT1-MUT-GFP. (G) After treating GC1 cells with GFP, STAT1-WT-GFP, and STAT1-MUT-GFP for 48 hours, dual-luciferase reporter assays were performed to validate the luciferase activities of the empty vector (EV), *Ccl2*-promoter-WT, and *Ccl2*-promoter-MUT reporter constructs. (H) After treating GC1 cells with GFP, STAT1-WT-GFP, and STAT1-MUT-GFP for 48 hours, dual-luciferase reporter assays were performed to validate the luciferase activities of the empty vector (EV), *Ccl5*-promoter-WT, and *Ccl5*-promoter-MUT reporter constructs. For E-H, n = 3 per group. Each experiment was independently repeated three times. The relevant p-values had been marked in the figures.


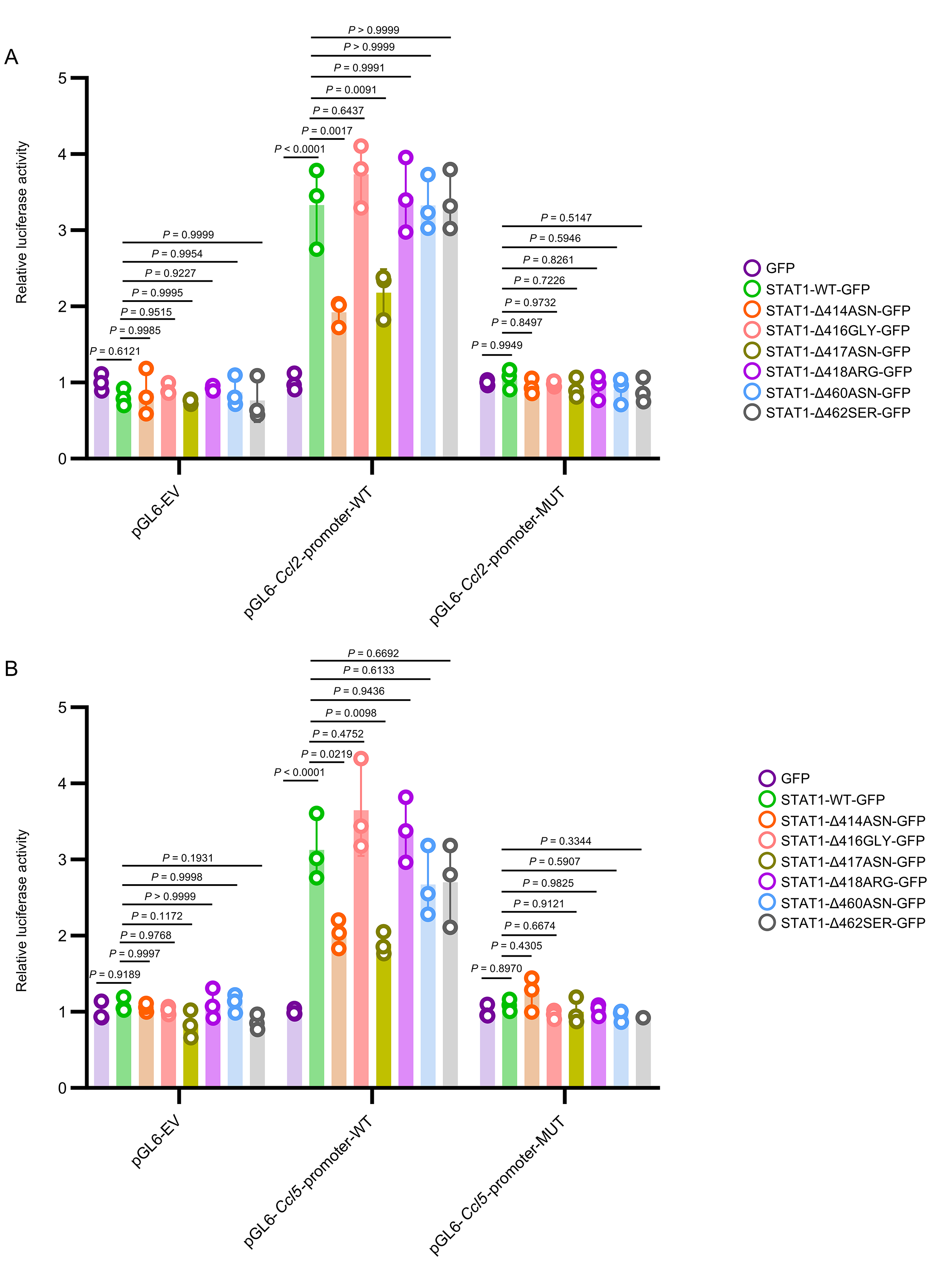


**Supplementary Figure 8** Identification of Key STAT1 Binding Regions Required for Ccl2 and Ccl5 Promoter Activation. (A) *Ccl2* and *Ccl5* shared six common binding sites in the STAT1-DBR region. Deletion mutants were generated for each individual site. After treating GC1 cells with GFP, STAT1-WT-GFP, and the respective deletion mutants for 48 hours, dual-luciferase reporter assays were performed to validate the luciferase activities of the empty vector (EV), *Ccl2*-promoter-WT, and *Ccl2*-promoter-MUT reporter constructs. (B) After treating GC1 cells with GFP, STAT1-WT-GFP, and the respective deletion mutants for 48 hours, dual-luciferase reporter assays were performed to validate the luciferase activities of the empty vector (EV), *Ccl5*-promoter-WT, and *Ccl5*-promoter-MUT reporter constructs. For A-B, n = 3 per group. The relevant p-values had been marked in the figures.


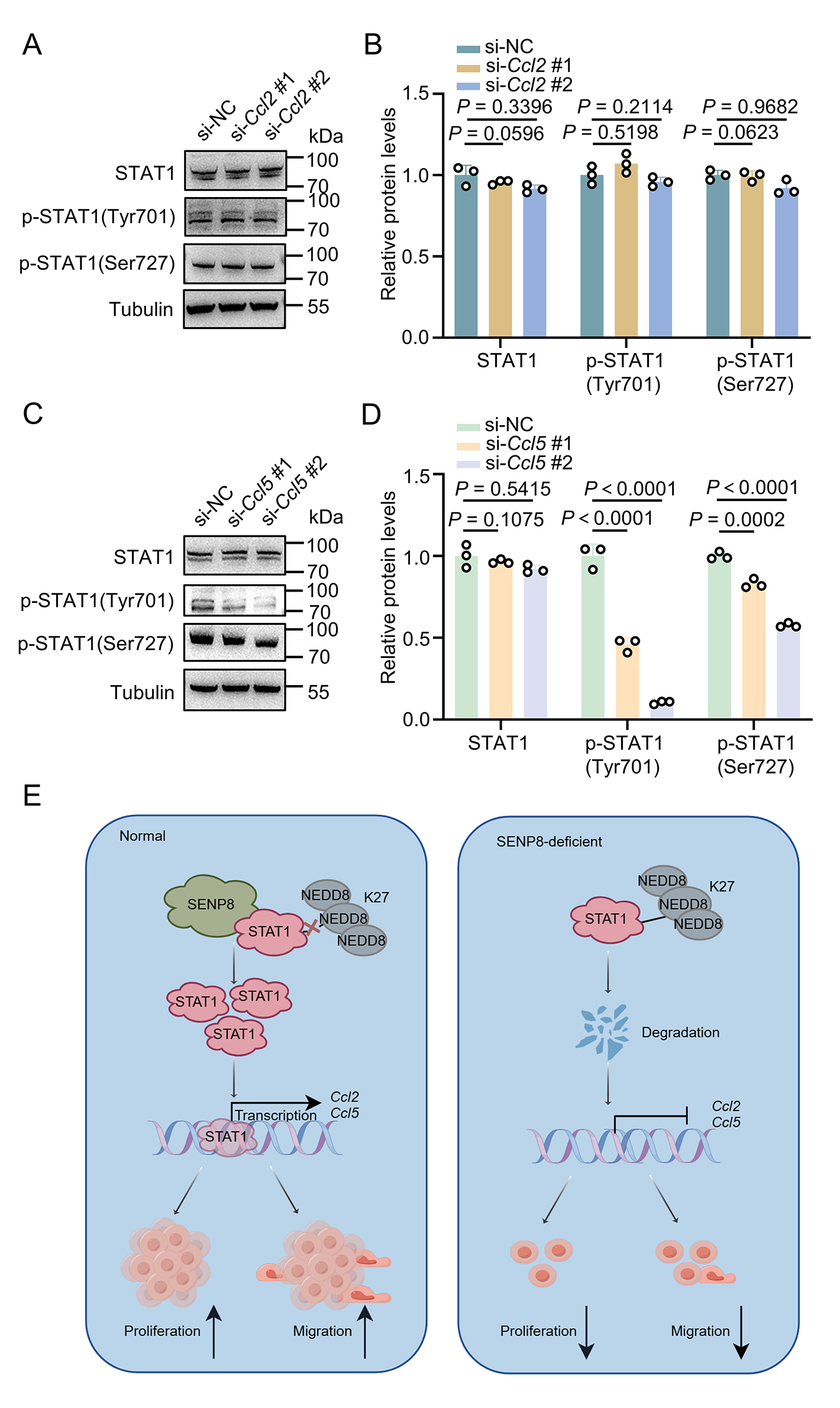


**Supplementary Figure 9** CCL5, but Not CCL2, Maintains STAT1 Phosphorylation in the SENP8–STAT1 Axis. (A,B) Changes in protein levels of STAT1, p-STAT1 (Tyr701), and p-STAT1 (Ser727) following *Ccl2* knockdown, n = 3 per group. (C,D) Changes in protein levels of STAT1, p-STAT1 (Tyr701), and p-STAT1 (Ser727) following *Ccl5* knockdown, n = 3 per group. (E) Proposed mechanism by which SENP8 regulates GC1 cell proliferation and migration. Each experiment was independently repeated three times. The relevant p-values had been marked in the figures.
